# Maternal Consumption of Non-Nutritive Sweeteners during Pregnancy Is Associated with Alterations in the Colostrum Microbiota

**DOI:** 10.3390/nu15234928

**Published:** 2023-11-26

**Authors:** Alejandro Tapia-González, Juan Manuel Vélez-Ixta, Nallely Bueno-Hernández, Alberto Piña-Escobedo, Jesús Carlos Briones-Garduño, Leticia de la Rosa-Ruiz, José Aguayo-Guerrero, Viridiana M. Mendoza-Martínez, Lenin Snowball-del-Pilar, Galileo Escobedo, Guillermo Meléndez-Mier, Lucía A. Méndez-García, Jaime García-Mena, Marcela Esquivel-Velázquez

**Affiliations:** 1 Laboratorio de Proteómica e Inmunometabolismo, Hospital General de México “Dr. Eduardo Liceaga”, Mexico City 06720, Mexico; alejandro_tapia@ciencias.unam.mx (A.T.-G.); nallely_bh5@yahoo.com.mx (N.B.-H.); benitojuarezgarciam@hotmail.com (L.S.-d.-P.); gescobedog@msn.com (G.E.); lucia.angelica86@gmail.com (L.A.M.-G.); 2 Departamento de Genética y Biología Molecular, Centro de Investigación y de Estudios Avanzados del Instituto Politécnico Nacional, Mexico City 07360, Mexico; juan.velez@cinvestav.mx (J.M.V.-I.); apinae@cinvestav.mx (A.P.-E.); 3 Servicio de Ginecología, Hospital General de México “Dr. Eduardo Liceaga”, Mexico City 06720, Mexico; 4 Banco de Leche Humana y Lactancia, Hospital General de México “Dr. Eduardo Liceaga”, Mexico City 06720, Mexico; 5 Facultad de Salud Pública y Nutrición, Universidad Autónoma de Monterrey, Monterrey 64460, Mexico; melendez651@gmail.com

**Keywords:** non-nutritive sweeteners, microbiota, colostrum, human milk, high-throughput nucleotide sequencing

## Abstract

Non-nutritive sweeteners (NNSs) provide a sweet taste to foods and beverages without significantly adding calories. Still, their consumption has been linked to modifications in adult’s and children’s gut microbiota and the disruption of blood glucose control. Human milk microbiota are paramount in establishing infants’ gut microbiota, but very little is known about whether the consumption of sweeteners can alter it. To address this question, we sequenced DNA extracted colostrum samples from a group of mothers, who had different levels of NNS consumption, using the Ion Torrent Platform. Our results show that the “core” of colostrum microbiota, composed of the genera *Bifidobacterium*, *Blautia*, *Cutibacteium*, *Staphylococcus*, and *Streptococcus*, remains practically unchanged with the consumption of NNS during pregnancy, but specific genera display significant alterations, such as *Staphylococcus* and *Streptococcus*. A significant increase in the unclassified archaea *Methanobrevibacter* spp. was observed as the consumption frequency of NNS increased. The increase in the abundance of this archaea has been previously linked to obesity in Mexican children. NNS consumption during pregnancy could be related to changes in colostrum microbiota and may affect infants’ gut microbiota seeding and their future health.

## 1. Introduction

High-sugar diets are linked to health problems such as the development of overweight and obesity, cardiovascular problems, and diabetes, among others [1]. As an alternative to sugars, non-nutritive sweeteners (NNSs) are widely used to preserve sweetness without significantly increasing the caloric content of foods and beverages [2]. NNSs are food additives approved to be consumed by the general public, including children and pregnant women [3]. However, NNS consumption has been associated with developing the same diseases related to high-sugar diets in adults [4,5]. An increasing amount of evidence demonstrates that the consumption of NNSs leads to alterations in the gut microbiota in both murine models and humans and these changes in microbiota are directly related to disruptions of blood glucose control [6,7,8,9].

Furthermore, NNS consumption has been associated with increased BMI and a higher risk of cardiometabolic diseases [10]. Recent studies show that the consumption of NNSs during pregnancy is associated with a higher body mass index in one-year-old infants [11,12] and with shifts in their gut microbiota (in murine models), such as an increase in firmicutes and a decrease in *Akkermansia muciniphila* [13]. NNS consumption during pregnancy is widespread; one out of every three pregnant women consumes NNSs on a daily (13.1%) or weekly (22.4%) basis, while the remaining 61.1% do so occasionally or never [14]. In addition, NNSs can be found in human milk when consumed by lactating women [15,16]. Human milk contains its own microbiota, which is crucial for establishing infants’ gut microbiota [17], but its relationship with the consumption of NNSs is rarely studied. Infants acquire their microbiota from maternal microbiota [18,19], birth mode [20], and feeding type (human milk or formula milk). Human milk microbiota can originate from the mother’s gut microbiota through the entero-mammary pathway and from the oral microbiota of the infant [21]. Considering the effects of NNS consumption on gut microbiota, such as the higher risk of overweight and metabolic diseases in NNS consumers and the fact that the gut microbiota are implicated in the origin of milk microbiota through the entero-mammary pathway, this work aims to explore whether the consumption of NNSs during pregnancy could be related to changes in the colostrum microbiota in a sample of Mexican women.

## 2. Materials and Methods

### 2.1. Study Design and Ethical Considerations

This was a cross-sectional study conducted from January 2018 to December 2019 at the Gynecological Unit of the Hospital General de México “Dr. Eduardo Liceaga” (HGMEL) in Mexico City.

### 2.2. Selection and Evaluation of Patients

The sample consisted of 168 in-labor healthy women between 18 and 40 years old who were invited to participate at their arrival to HGMEL with a gestational age of >35 weeks which was measured by ultrasound (USG) or estimated by last menstrual period (LMP). The objectives and procedures of the study were thoroughly explained to them. Women with diabetes mellitus, gestational diabetes, hypertension, thyroid disorders, autoimmune diseases, or other acute (active infections of any kind requiring the use of antibiotics, insufficiencies such as cardiac, hepatic, renal, etc; cholestasis of pregnancy) or chronic health problems were excluded from the study. Also, participants with no colostrum sample were eliminated, as well as those whose newborns had any health problem that required neonatal intermediate or intensive care unit attention. The mother’s height and weight were registered immediately after delivery. A clinical questionnaire, including the use of antibiotics over the last six months, was administered. A pediatrician examined all of the newborns after birth, clinically determined the newborns’ health status, and calculated the gestational age using the Capurro method [22]. The mothers’ and newborns’ body compositions were assessed using the RJL Quantum IV System (RJL Systems Inc., Clinton Township, MI, USA) after birth. The Hospital General de México is a public hospital where breastfeeding is promoted; mothers and their newborns cohabit while in the hospital. All the mothers included in this study and their newborns were healthy, and all were given an orientation on breastfeeding. All newborns, at least until discharge from the hospital, were breastfed.

### 2.3. Non-Nutritive Sweeteners Consumption Assessment

Two previously validated questionnaires were administered to all of the women included in the study. These were (1) The Food Frequency Questionnaire of products with NNS (FFQIS) [23], and (2) a 24-h dietary recall (24HR) [24]. The FFQIS explored the consumption of all commercially available products in Mexico in 2017 containing at least one NNS. In the 24HR dietary recall, each woman freely described their food intake over the previous 24 h; this questionnaire was administered after admittance but before delivery (which usually occurred within the following 12 h after admission). Two nutritionists evaluated both questionaries and calculated the macro and micronutrients consumed using the nutre.in software (https://nutre.in/, accessed from January to March 2023).

Participants were categorized into four groups according to the statistical quartiles of the consumption frequency of products with NNS: Q1 (<4 times/week), Q2 (4 to <8 times/week), Q3 (8 to <16.5 times/week), and Q4 (≥16.5 times/week).

### 2.4. Sample Collection and Processing

Trained personnel from the hospital’s human milk bank manually collected the colostrum samples in sterile glass bottles within 24–48 h after delivery. The breast was cleaned with sterile gauze soaked in sterile water and the personnel used sterile gloves during the extraction. Samples were collected in the morning, between 10 and 12 h, and a maximum of 3 mL was obtained from each participant. Colostrum samples were immediately stored at −20 °C for short-term storage at the hospital’s human milk bank per their protocols and then at −70 °C until use.

### 2.5. DNA Extraction

DNA was extracted from colostrum samples using the FavorPrep^TM^ Milk Bacteria DNA Extraction Kit (Favorgen^®^, Ping Tung, Taiwan) following the manufacturer’s instructions. The DNA was quantified using the DS-11FX spectrophotometer (DeNovix, Wilmington, DE, USA).

### 2.6. Preparation of the 16S rDNA Library and High-Throughput Sequencing

For each sample, a PCR reaction was carried out in order to amplify the 16S rRNA gen V3 hypervariable region while tagging it with different barcodes (1 to 100) for identification during sequencing; this was made following a previous report [25]. All reactions were performed on a final volume of 20 µL with 200 µM dNTPs, 2 mM MgCl_2_, 0.5 µM of each primer, 0.02 U/µL Phusion^TM^ high-fidelity DNA polymerase (ThermoScientific, 1X Phusion HF Buffer and DNA (10 ng). The quantity of DNA was increased for samples that did not amplify with 10 ng until amplification was observed (max. 240 ng). The thermocycling was made using a miniPCR^®^ thermal cycler (miniPCR bio™, Cambridge, MA, USA) with 98 °C for 3 min, followed by 30 cycles (98 °C for 12 s, 62 °C for 15 s, and 72 °C for 10 s), ending with a final extension at 72 °C for 5 min. Agarose gels (2%) were used to observe and quantify each sample’s expected amplicon (~281 bp) by densitometry. The library for sequencing was prepared by mixing equal mass amounts of each barcoded amplicon (1–100).

### 2.7. High-Throughput DNA Sequencing

Once the V3-16 rRNA gene library amplicons were mixed, a highly sensitive 2% agarose gel stained with SYBR GOLD DNA (E-Gel^TM^ EX, 2%, Invitrogen^TM^, Cat. G401002, Waltham, MA, USA) was used to purify the final library. The DNA library concentration and final size fragment were measured with the 2100 Bioanalyzer Instrument (Agilent Technologies, Santa Clara, CA, USA) fragment analyzer; the resulting average size of the library was 263 bp. According to the manufacturer’s instructions, emulsion PCR was carried out using Ion OneTouchTM 200 Template Kit v2 DL (Life Technologies, Carlsbad, CA, USA). The amplicon was enriched using Ion OneTouch ES ionic spheres (Life Technologies, Carlsbad, CA, USA). Sequencing was performed using the Ion 318 Kit V2 Chip (Cat. 4488146, Life Technologies, Carlsbad, CA, USA) and the Ion Torrent PGM system v4.0.2. After sequencing, the readings were filtered by the PGM software to remove the polyclonal (homopolymers > 6) and low-quality sequences (quality score ≤ 20).

### 2.8. Taxonomic Assignment and Bacterial Diversity

Amplicon sequence variants (ASV) were determined from reads that met the quality criteria using the QIIME2-2022.2 pipeline [26] using the dada2 plugin with the option “–p-trunc-len” set to 173 nucleotides. Representative sequences were taxonomically annotated with Silva 138 database release using the pre-formatted files provided by the QIIME website [27] with 97% percentage of identity. Further analyses were performed with R 4.3.1 Beagle Scouts [28] in RStudio 2023.6.0.421 IDE [29]. Data were imported into R with qiime2R 0.99.6 package [30], phyloseq 1.44.0 package [31] was used as it provides a data structure and useful basic function for the analysis of microbial communities. For intra-sample diversity Observed, Shannon, Simpson, InvSimpson, and Fisher indexes were calculated. Analysis of the inter-sample diversity was carried out with UniFrac distance, and non-metric multidimensional scaling (NMDS) ordination with vegan 2.6–4 package [32], the optimal number of clusters and partitioning around medoids was performed with fpc 2.2–10 [33]. The core microbiota heat map (60% prevalence, 10% detection) was obtained with microbiome 1.22.0 [34]. The package heatmaply 1.4.2 [35] was used to elaborate the heatmaps. Differential abundance analysis was performed with DESeq2 1.40.2 [36], data were managed with tydiverse 2.0.0 [37] and multipanel figures were elaborated using ggpubr 0.6.0 [38] and scales 1.2.1 [39].

### 2.9. Statistical Analysis

Epidemiological and clinical data are presented as mean± standard deviation, median [IQR], where the IQR (interquartile range) is presented as [first quartile; third quartile]; or as frequencies and percentages. The distribution of data was assessed using the Kolmogorov–Smirnov test. ANOVA or Kruskal–Wallis tests were used to compare between the four groups. The Chi-squares test was used to compare qualitative variables among groups and clusters. The Mann–Whitney U test was used for comparing pairs of groups, and Spearman’s rho test was used for correlations. The strength of the correlations was classified according to [40]. The statistical software SPSS v25 was used, considering a *p* < 0.05 statistically significant. All reported *p* are two-tailed.

## 3. Results

### 3.1. Study Population

A total of 168 women met the inclusion criteria and were invited to participate in the study, from which 82 women accepted to donate colostrum samples. Most of them (98.7%) lived in Mexico City and its metropolitan area (Figure S1), where Iztapalapa is the most frequent municipality of residence (Figure S2). Mexico City is 2240 m over sea level and has 9,209,944 inhabitants, with a population density of 6163.3 per km^2^ [41]. It is divided into 16 municipalities, from which Iztacalco, Cuauhtémoc, Benito Juárez, and Iztapalapa are the most densely populated municipalities with over 16,000 habitats per km^2^ [42].

The average age of the women was 24 ± 5.14 years (median 23 years), with pregnancies of 38.8 ± 0.2 weeks that gave birth to babies with estimated gestational age by the Capurro method of 39.54 ± 0.24 weeks.

#### 3.1.1. Descriptive Statistics

The participants’ consumption frequency of products with NNS was 12.72 ± 1.32 times/week, with a median of 9.5 (range 0–49). Considering the wide range in the frequency of consumption of NNSs, four groups were generated (Q1, Q2, Q3, and Q4), which correspond to the statistical quartiles of this variable. We decided to perform such statistical division to avoid the arbitrary conformation of groups, considering that this type of grouping has been used in other studies on the consumption of NNSs [43]. The demographic and clinical characteristics of the women in each group are presented in Table 1. The four groups were homogeneous, although we found significant differences between the four groups in the average number of children, the first child rate (number of women for which this was their first child/n), the age of menarche (which was only different between Q2 and Q4 (U = 662, *p* = 0.020)), and the height. A fair negative correlation was identified between the NNS consumption frequency and the number of children (r_s_ = −0.376, *p* = 0.001) and a poor negative correlation with the age of menarche (r_s_ = −0.257, *p* = 0.020). Likewise, a poor negative correlation was identified between the frequency of NNS consumption with the gestational age (using the Capurro method, r_s_ = −0.228, *p* = 0.041) and with the newborn’s length (r_s_ = −0.232, *p* = 0.041) and weight (r_s_ = −0.225, *p* = 0.048), although the gestational age calculated with USG/LMP did not correlate with the frequency of NNS consumption. The women in the Q4 group were the youngest, while the Q1 group displayed the highest average age (U = 328.5, *p* < 0.001).

#### 3.1.2. Diet Characteristics

The macro- and micronutrient intake of the women included in the study were obtained from the 24HR dietary recall and compared between the four groups (Table 2). No differences were found between the groups except for sugar intake, which was higher in the Q1 group than in the Q2 group (U = 660, *p* = 0.014) and the Q3 group (U = 487.5, *p* = 0.006), but similar to the Q4 group (U = 543.5, *p* = 0.129). In addition, potassium intake was only different between Q2 and Q4 groups (U = 682, *p* = 0.010). A poor negative correlation was found between the frequency of NNS consumption and daily sugar intake (r_s_ = −0.231, *p* = 0.037), but no correlation was found with potassium intake. However, with statistical significance, the four groups were not different in other macro- and micronutrients, the daily intake of calcium was higher in the Q2 and Q3 groups compared to the Q4 group (U = 673, *p* = 0.047 and U = 484, *p* = 0.014, respectively) and in the Q3 vs. Q1 group (U = 557.5, *p* = 0.043). The glycemic index (GI) was also higher in the Q1 group compared to the Q2 (U = 713, *p* = 0.046) and Q3 groups (U = 564.5, *p* = 0.051). Likewise, a high percentage of participants in every group showed lower-than-recommended daily intakes of several macro- and micronutrients. Only protein deficiency was different between the groups (*p* = 0.006), in which women in the Q4 group displayed the highest frequency of deficiency (90.5%). As for cholesterol intake, fewer women showed excessive intake (>300 mg per day), according to the National Cholesterol Education Program Adult Treatment Panel III (ATPIII) [44].

### 3.2. Colostrum Microbiota

#### 3.2.1. Alpha and Beta Diversity of Colostrum Microbiota Are Not Related to the Consumption of Non-Nutritive Sweeteners

**Figure 1:**
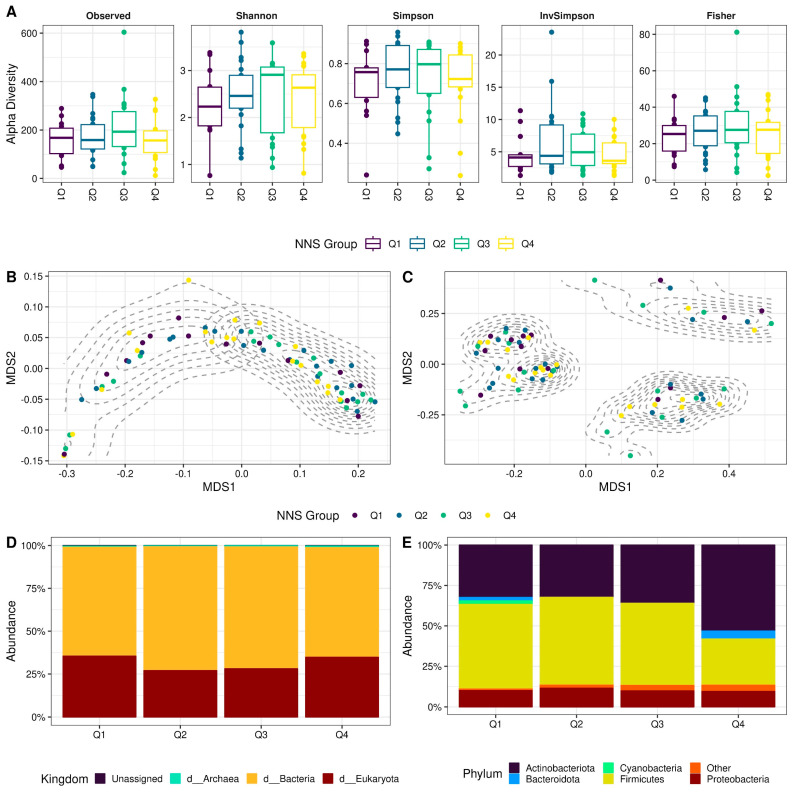
(**A**) Alpha diversity indexes represented by boxplots. The Y−axis represents alpha diversity measures, and the X−axis indicates the NNS groups, which are also highlighted with the box color. Kruskal−Wallis or analysis of variance was applied depending on the distribution, but no statistical difference was found (Table S2). (**B**,**C**) Non-metric multidimensional scaling (NMDS) scatter plots representing weighted and unweighted UniFrac distance metrics, respectively. Dashed grey lines represent the density of the optimal number of clusters (Figure S3); statistical differences were found for the cluster but not for the experimental groups (Figure S4). (**D**,**E**) Stacked bar plots representing the compositional microbiota at kingdom and phylum levels, respectively. The Y−axis indicates the relative abundance of taxa, and the X−axis shows the experimental groups; colors highlight the taxa. Kruskal−Wallis was applied, but no significant difference was found among groups (Tables S3 and S4).

The characterization of the alpha diversity in the Q1, Q2, Q3, and Q4 groups did not show differences in the Shannon, Simpson, invSimpson, or Fisher indexes (Figure 1A). The beta diversity analysis showed statistically significant clusters unrelated to the experimental groups: two for the weighted UniFrac distance metrics (Figure 1B) and three for the unweighted UniFrac distance metrics (Figure 1C). Since the optimal number of clusters was unrelated to the NNS consumption groups, different clinical and epidemiological variables were explored concerning the weighted and unweighted calculated clusters (Supplementary Table S1). The place of residence was also unrelated to the identified clusters (Figures S5 and S6), but the use of antibiotics was significantly different for the unweighted clusters (Figure S7), which can partially explain the observed clusters.

#### 3.2.2. The Composition of the Colostrum Microbiota Appears Unrelated to the Consumption of Non-Nutritive Sweeteners at the Kingdom and Phylum Levels

The eukaryote/bacteria ratio was analyzed to evaluate the proportion of Bacteria in colostrum samples. The relative abundance of eukaryote was similar in the Q1 and Q4 groups, appearing higher in both groups than in the Q2 and Q3 groups (Figure 1D). The composition of colostrum microbiota was evaluated at the phylum level; the relative abundance of proteobacteria was similar in the four groups, and the relative abundance of actinobacteria and Bacteroidota seemed higher in the Q4 group than in the other groups (Figure 1E); however, these differences were not statistically significant (Table S4).

**Figure 2:**
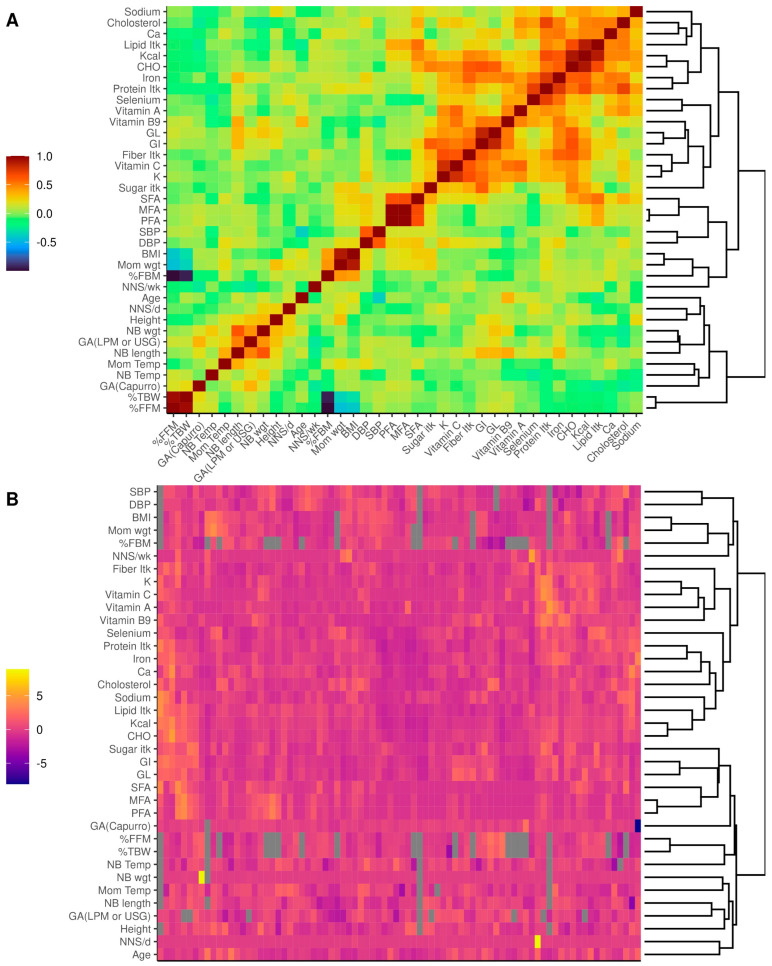
(**A**) Spearman correlation heatmap of continuous metadata measured in the study. Variable names are indicated in the X and Y axes, the right dendrogram shows the optimal leaf ordering (OLO) of metadata, and the left color bar indicates a strong positive correlation (1), no correlation (0), or strong negative correlation (−1). (**B**) Distribution heatmap of continuous metadata measured in the study. Variable names are indicated in the y−axis, the x side of the matrix represents each sample included in the study, the right dendrogram shows the OLO of samples and metadata, and the left color bar indicates a centered t−distributions of the metadata. Ca: calcium; Itk: intake; CHO: carbohydrates; GL: glycemic load; GI: glycemic index; SFA: saturated fatty acids; MFA: monounsaturated fatty acids; PFA: polyunsaturated fatty acids; DBP: diastolic blood pressure; SBP: systolic blood pressure; BMI: body mass index; Mom: mother; wgt: weight; % FBM: percentage of fat body mass; NNS: non−nutritive sweeteners; wk: week; d: day; NB: newborn; Temp: body temperature in °C; GA: gestational age; LMP: last menstrual period; USG: ultrasonography; % TBW: percentage of total body water; % FFM: percentage of lean body mass.

To identify whether some variables could be possible confounders, we first explored the co-occurrence of the continuous variables measured in the study by Spearman’s rank correlation to identify groups of features that might be implicated in the microbiota composition (Figure 2A); it was observed that variables related to nutrient consumption were correlated among themselves. However, apart from that, no other correlated variables were found. Likewise, we explored the distribution of the variables among women with a heatmap (Figure 2B) in which it can be observed that the samples are homogeneous since no evident groups were formed regarding the variables explored.

#### 3.2.3. Non-Nutritive Sweetener Consumption during Pregnancy Associated with Changes in Specific Genera of the Colostrum Microbiota

**Figure 3:**
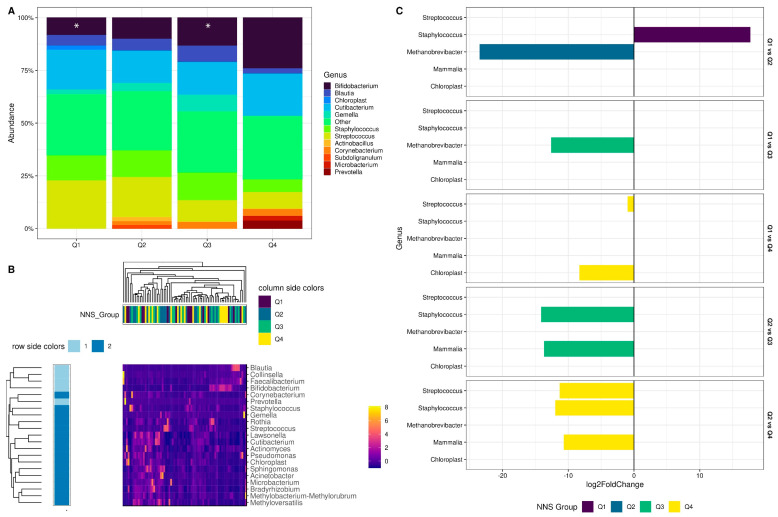
(**A**) Stacked bar plots representing the compositional microbiota at the genus level. The Y-axis indicates genus relative abundance, the X-axis shows the experimental groups, and colors highlight the genus. According to the Kruskal–Wallis test, asterisks were added to represent the statistical difference found in Bifidobacterium between Q1 and Q3 groups (See Figure S8 and Table S5). (**B**) Core genus heatmap at 60% prevalence and 1% detection cutoffs. The matrix left column indicates genera of the core microbiota, row side colors suggest the presence of two bacterial clusters calculated with partitioning around medoids (PAM) clustering method, column side colors indicate the experimental group of the samples, the right color bar indicates relative abundance levels scaled at the rows. (**C**) Bar plot indicating differential abundance analysis of bacteria with DESeq2. The left Y-axis indicates genus, the right Y-axis shows pairwise comparisons where bacteria with *q*-values < 0.05 were found, and the X-axis indicates log2FoldChange; color was added to the bars highlighting the NNS groups to facilitate interpretation (Table S6).

We then explored the composition of colostrum microbiota at the genus level. We found that the relative abundance of *Bifidobacterium* increased as the frequency of consumption of NNSs increased (from Q1 to Q4); however, these differences were not statistically significant according to the Kruskal–Wallis test (Table S5), except for the comparison between Q1 and Q3 (Figure 3A). The relative abundance of *Blautia* and *Staphylococcus* decreased, and *Prevotella* increased as the frequency of consumption of NNSs increased (Figure 3A). Nonetheless, the differences between the groups were not statistically significant. The core microbiota heatmap with the most abundant genera showed that the NNS groups did not cluster together (Figure 3B), which suggests that these genera are unrelated to the frequency of consumption of NNSs. However, the differential abundance analysis with DESeq2 revealed that the specific genera differed between the NNS consumption groups. *Staphylococcus* was 17.7 times higher in Q1 vs. Q2, while *Methanobrevibacter* was 23.49 times higher in Q2 vs. Q1 and 12.6 times higher in Q3 vs. Q1. Other genera, such as *Streptococcus*, also showed differences, being higher in Q4 vs. Q1 and Q2; *Staphylococcus* was more than ten times higher in Q3 and Q4 vs. Q2. The relative abundance of DNA of mammalian origin also showed differences, being more than ten times lower in Q3 and Q4 vs. Q2, possibly indicating a lower bacteria abundance in the colostrum as the frequency of NNS consumption increased. A bacterial genus, like *Chloroplast*, was also higher in Q4 vs. Q1 (Figure 3C, Table S6).

## 4. Discussion

Human milk microbiota are essential for establishing a healthy infant gut microbiota [46]. Maternal factors such as NNS consumption have been reported to influence their offspring’s gut microbiota and affect their future health in murine models [44,45]; however, the information is rarely reported in humans. In this cross-sectional study, we assessed the microbiome composition of the colostrum, focusing on the frequency of consumption of products containing NNSs during pregnancy. We found that the most abundant bacterial phylum in the colostrum were actinobacteria, firmicutes, and proteobacteria, with no significant differences in their relative abundance related to the frequency of consumption of products with NNSs, from which we can assume that the consumption of NNSs did not alter the composition of the colostrum microbiota at the phylum level. It has been reported in other studies that a higher Firmicutes/Bacteroidetes ratio is associated with obesity [47]; in this context, women with a higher NNS consumption could also exhibit different Firmicutes/Bacteroidetes ratios, but this was not the case. On the other hand, the Proteobacteria phylum is characterized by many bacteria commonly associated with inflammation [48,49], like salmonella or Escherichia, thus suggesting alterations in the microbiota; this was also not the case. These phyla have also been reported to be the predominant taxa in human milk samples in the Mexican population [25]; however, the relative abundances we report here appear different from those previously reported, probably due to methodological differences such as the time window for the collection of human milk (0–48 h postpartum in our study vs. 1–6 days postpartum) or the inclusion criteria (we did not exclude probiotics or antibiotics use in the third trimester).

The most abundant genera in our study were *Bifidobacterium*, *Blautia*, *Chloroplast* (not the plant chloroplast, but a bacterial sequence similar to Chloroplast; evolutionarily chloroplasts descend from bacteria and a high degree of homology in the 16S genes have been reported [50]), *Gemella*, *Staphylococcus*, *Streptococcus*, *Actinobacillus*, *Corynebacterium*, *Subdoligranulum*, *Micobacterium*, *Cutibacterium,* and *Prevotella*, which is consistent with what has been reported elsewhere [51]. The relative abundances of these genera were similar between the quartiles of NNS consumption frequency; however, *Bifidobacterium* seemed to increase as the frequency of NNS consumption increased. *Bifidobacterium* is an early gut colonizer in infants [52], and it is known as a beneficial bacterium due to the modulation of host immune responses (for instance, decreased incidence of allergies) [53] and protection against infectious diseases [54]. Human milk oligosaccharides (HMOs) promote the growth of *Bifidobacterium* in the infant’s gut, and human milk is also the source of *Bifidobacterium* to the infant. It has been reported that the NNS aspartame increases *Bifidobacterium* growth in an in vitro fermentation experiment with fecal samples [55]; nonetheless, this is an unlikely scenario in vivo since aspartame is rapidly metabolized upon ingestion into phenylalanine, aspartic acid, and methanol, thus not reaching the gut microbiota. On the other hand, another widely used NNS, sucralose, has been reported to reduce the abundance of *Bifidobacteria* in the guts of rats [56]. The abundance of *Bifidobacteria* in human milk is variable, ranging from 0 to 16% in human milk samples from 4 to 7 days after birth [57], so it is possible that the increase in *Bifidobacterium* we observed as the frequency of NNS consumption increased is likely due to the intrinsic differences of the individuals involved in the study. Another explanation could be that NNSs may have differential effects on the microbiota of different human body sites since each site has a unique ecological niche, and the complex interactions occurring in each site still remain largely unknown. Furthermore, NNSs may affect the permeability of the gut barrier, which could, in turn, affect gut bacteria translocation and migration to the mammary gland in the entero-mammary pathway. Experiments with the Caco-2 epithelial cell line have shown that aspartame, saccharin, and sucralose increase epithelial barrier permeability [58], but the effect of NNS on the gut epithelial barrier of pregnant women, which is already more permeable than normal [59], is unknown. The Antibiotic treatment during pregnancy or lactation has also been reported to affect the abundance of *Bifidobacteria* in human milk [60], although, in our results, no differences were found between the four groups of NNS consumption regarding the use of antibiotics over the six months previous to delivery. However, we did not register the antibiotic therapy’s time, type, and length.

We did not observe any differences between the NNS groups in other abundant genera, but we did identify differences in some genera between specific groups using DESeq2. For instance, *Staphylococcus* was significantly higher in the Q1, Q3, and Q4 groups in comparison to the Q2 group, suggesting that this genus does not change as NNS consumption increases, or that it does so only with the quantities of NNS ingested in the Q2 group. Several possible explanations arise: the first is that Q2 happened to group samples with lower staphylococcus than the other groups in a random event; another is that women in the Q2 group could have consumed more of a certain type of NNS or been exposed to another substance not assessed in the study. Likewise, *Streptococcus* abundance was higher in the Q4 group in comparison to the Q2 and Q1 groups, which suggests that its relative abundance increases with the frequency of NNS consumption. In both cases, the differences in the abundance of both genera did not seem to follow a clear pattern as the frequency of consumption of NNS increased. It is possible that both *Staphylococcus* and *Streptococcus* may present an erratic behavior with NNS, in which only a specific amount of exposure to NNS is associated with the changes in these genera in the colostrum samples, but at different exposures, the effect is not present anymore. In a previous study, we performed a clinical trial with two doses of NNS sucralose (48 mg and 96 mg) which were given on a daily basis to healthy volunteers for ten weeks. The most significant effects on the insulin and glucose responses were obtained with the lower dose (48 mg) but not with the 96 mg dose [8]. It has been reported that insulin and glucose responses are modified with the consumption of NNS in a way that depends on the changes produced by NNS in the gut microbiota [7]. However, it has also been reported that some individuals resist NNS’s changes in the gut microbiota [7]. We do not know whether certain groups could have a higher proportion of such individuals nor to which NNS they may have such resistance.

Regarding archaea, *Methanobrevibacter* was another genus that showed statistically significant changes between the NNS consumption groups. *Methanobrevibacter* is a gut-associated archaeon that has been associated with weight regulation, and that is also present in human milk. Furthermore, *Methanobrevibacter* in human milk is essential in seeding the infant gut. *Methanobrevibacter smithii* is the most widely described human methanogenic archaea and is present in healthy, lean human adults, but it is decreased in individuals with obesity [61]. In contrast, other studies in mouse models associate the abundance of *M. smithii* with increased adiposity due to enhanced dietary fiber usage [62].

Moreover, in children, the presence of *M. smithii* is associated with an increased risk of overweight and higher weight z-scores [63]. In addition, a published report describes an increased abundance of unclassified *Methanobrevibacter* spp. in 10-year-old Mexican children affected by overweight and obesity [64]. Our results show that *Methanobrevicater* increased in the Q2 and Q3 groups compared to the Q1 group, which suggests that the abundance of this genus increases as the frequency of consumption of products with NNS increases. It is an interesting finding considering that several studies associate the consumption of NNSs during pregnancy and lactation with changes in the gut microbiota of the offspring and with a higher risk of overweight, obesity, type 2 diabetes, and metabolic syndrome later in life [65]. In epidemiological studies, the consumption of artificially sweetened beverages (ASB) during pregnancy has been associated with preterm delivery [66,67,68], higher BMI and overweight at the age of 1 year [11], and with an increased risk of asthma in childhood [69]. Considering our work is a transversal study, we did not follow the infants after pregnancy, so we could not correlate the abundance of *Methanobrevicater* in the colostrum samples with their BMI later in life. However, we did notice a negative correlation between the frequency of consumption of products with NNSs and gestational age using the Capurro method and with the height and weight of the newborn, but not with the gestational age, with LMP/USG, even though the selection criteria of this study had a very narrow window (more than 37 weeks of pregnancy) of inclusion. In a not-yet-published work, Garcia-Mena’s group found that *Methanobrevibacter* is differentially abundant in the colostrum in comparison to the feces of newborns, suggesting that at the time of assessment, these archaea may not have fully colonized and proliferated in the gut of the infants (unpublished data), so, although we did not assess the newborn’s gut microbiota, something similar it is to be expected considering that both studies were conducted within the same Mexican population. More research is needed to assess whether the abundance of *Methanobrevibacter* in the colostrum is associated with a higher BMI later in life, especially considering that childhood obesity is a growing worldwide health problem [70] that is directly linked to adult obesity and increases the risk of cardiovascular and metabolic problems in adulthood [71,72].

On the other hand, the analysis of the alpha diversity of the microbiome showed that the groups of frequency of consumption of NNSs were similar; nonetheless, the analysis of the beta diversity revealed two significantly different clusters with the weighted UniFrac and three clusters with the unweighted UniFrac, none of which were related to the groups of frequency of consumption of NNSs. Several variables were analyzed to try to identify some that could explain the clustering, from which the use of antibiotics within the previous six months before delivery could partially explain the unweighted clusters but not the weighted clusters. The place of residence of the women included in the study was also explored since the geographical location of the participant women has been previously associated with differences in milk microbiota [73]. In our sample, the different clusters were not associated with this variable, even though the Mexico City municipalities have important socioeconomic differences [74].

Concerning the reported presence of Eukaryote in this study, it is important to note that when primers targeting the hypervariable region of the V3–V4 16S rRNA gene are used, the off-target amplification of human DNA is common, especially for samples that have a high abundance of human cells and low microbial biomass [75] such as the human milk, which contains different cell types derived from the breast (lactocytes, myoepithelial cells, progenitor cells, stem cells) and from the blood (immunological cells, hematopoietic stem cells) of the mother [76]. In addition, we did not find a statistically significant difference in the relative abundance of Eukaryote between the four NNS groups; in terms of relative abundances of Eukaryote, all groups seem to have the same proportions. In some cases, the relative abundance mean measure could be misleading because the mean is very sensitive to outliers; in the same sense, we opted to use the Kruskal–Wallis non-parametric test that compares the rank totals instead of the means. Nonetheless, DESEq2 (Figure 3C) highlights a higher abundance of an ASV identified as Mammalia in Q3 and Q4 with respect to Q2. The interpretation is this unique ASV could increase as NNS consumption increases.

Of note, the NNS consumption quartiles were clinically similar. No differences in body composition were found; likewise, all of the women included were healthy without diabetes, hypertension, or any metabolic diseases. This suggests that the frequency of consumption of products containing NNSs was not associated with a professional recommendation or personal desire to limit weight gain during pregnancy. Notably, the BMI average in all groups falls into the overweight category, even after correcting for postpartum weight. Most of the women included in his study just arrived at the HGMEL for delivery without receiving prenatal care in the hospital, or if they did, it was only in the second or third trimester, so the weight before pregnancy was unavailable.

Similarly, the diet composition of women in the four NNS groups was similar in macro and micronutrients, with some differences in potassium content that did not follow a trend as the frequency of NNS consumption increased. Also, sugar intake differed between the groups, being higher in Q1 (the group with the lowest frequency of NNS consumption) than in the other groups and lowest in the Q3 group. Daily sugar intake showed a poor negative correlation with the frequency of consumption of products containing NNS. in randomized controlled clinical trials (RCT), the administration of NNSs as a replacement for sugars in the diet has been shown to reduce daily sugar intake [77], which is consistent with what we have observed in this study. In addition, the comparison of the intake of calcium, cholesterol, SFA, and phosphorus between the groups resulted in a *p*-value between 0.05 and 0.1. This tendency will probably result in a statistically significant *p*-value increasing the sample size, although the sample size required for this is uncertain. Nevertheless, there was no tendency to increase or decrease as the consumption frequency of NNS increases from groups Q1 to Q4 in any of those variables, so we consider the tendency to be due to chance alone and not to the diet associated with the different NNS consumption groups. On the other hand, a very high percentage of women in all groups had a lower-than-recommended daily intake [45] of proteins, carbohydrates, sodium, potassium, calcium, vitamin A, vitamin B9 (folate), and vitamin C, and all women in all of the groups (100%) displayed a deficient intake of iron, phosphorus, and selenium. The very high frequency of deficient intake of these nutrients is worrying since the answers given in the 24HR questionnaire could be a reflection of the overall diet through pregnancy, but caution should be taken to interpret these results considering that the 24HR was administered to the women after admission to the hospital but before the birth of the baby; women were only admitted to the hospital in an established stage of labor, so before their admission, many of them may have been many hours in the latent stage of labor and not feeling very well, which may have affected their food intake.

It is important to mention that the women consumed products containing multiple NNSs, each in different combinations and amounts, so no specific NNS can be related to a particular behavior of the colostrum microbiota. In this study, no substantial differences were identified in the clinical characteristics between the four groups of NNS consumption that could be related to the observed differences in colostrum microbiota, although some variables were statistically different. For instance, the age of menarche was statistically different between the groups but with no clinical relevance. The number of children and the first child rate were also different between the groups and showed a negative correlation with the frequency of consumption of NNSs; the women who consumed NNSs more frequently were the ones who had fewer children, contrary to what one should expect; considering that the retention of some weight after each pregnancy is frequent [78], it would have made sense that the women with more children were also the women who consumed more NNSs in an attempt to gain less weight during their current pregnancy, however, that was not the case.

The consumption of NNSs has been associated with changes in gut microbiota in humans, particularly with the intake of saccharin, sucralose, and stevia [6,7,9]. Furthermore, in a murine model, the administration of the NNS stevia to dams during pregnancy and lactation affects the microbiome of the pups, even though NNSs were not administered directly to the offspring [79]. Likewise, the offspring of mice to whom NNS was administered during pregnancy and/or lactation showed changes in the gut microbiota and in the microbiota metabolites and downregulation of hepatic detoxification mechanisms [13]. The changes in colostrum microbiota between the NNS consumption groups can be explained considering that the milk microbiota originate partially from an entero-mammary pathway that transports bacteria from the mother’s gut to the mammary gland and that NNSs modify the gut microbiota and can be found in the human milk. We consider it positive that few differences were found in the colostrum’s “core” microbiota between the NNS groups; nonetheless, we did find some differences. To the best of our knowledge, this is the first study that assesses the microbiota profile of colostrum using high-throughput sequencing in relation to the frequency of NNS consumption, opening the possibility of the existence of an early link between the development of future obesity in children and changes in the microbiota of human milk. NNS consumption did not greatly impact human colostrum microbiota, but some individual features seemed to change as NNS consumption increased; however, some of these findings are inconclusive, as there is no straightforward pattern for them. With this work, we can conclude that NNS consumption during pregnancy could be related to changes in colostrum microbiota in a sample of Mexican women, particularly in the *Methanobrevibacter* genus. No changes were found in colostrum’s “core” microbiota, composed of the genera *Bifidobacterium*, *Blautia*, *Cutibacteium*, *Staphylococcus*, and *Streptococcus.* More studies in other populations and a bigger sample size are necessary to corroborate our findings. Likewise, the possible effects of these changes in the colostrum microbiota on the future health of infants need to be studied further.

## Figures and Tables

**Table 1:** Clinical characteristics of the subjects included in the study, shown in quartile groups of frequency of NNS consumption.

Variable	Q1	Q2	Q3	Q4	F^a^/H^b^/χ^2^	*p*
	<4 t/wk	4 to <8 t/wk	8 to <16.5 t/wk	≥16.5 t/wk		
	*n* = 16	*n* = 25	*n* = 20	*n* = 21		
Age, years	26[21;30]	23[20;23]	24[20;31]	20[18;26]	6.391	0.094 ^b,d^
Children, n	2[1;2]	2[1;2]	1[1;2]	1[1;1]	11.600	0.009 ^b,d,f^
First child rate, n (%)	4(25)	11(44)	11(55)	17(81)	12.442	0.006 ^c^
Menarche age, years	13[11;14]	13[12.8;14.3]	12.5[11;13]	12[11;12]	10.079	0.018 ^b^
Gestational age (Capurro), weeks	39.8[39;41.1]	40[39.2;41.1]	39.1[38.1;40.3]	39.5[38;40]	4.340	0.227 ^b^
Gestational age (USG/LMP), weeks	39.8[39.5;40.4]	39[37.8;40.1]	38.5[37.1;40.2]	39.1[36;39.6]	3.449	0.327 ^b^
Systolic pressure, mmHg	105[100;112.5]	110[100;120]	105[100;110]	100[100;110]	0.767	0.857 ^b^
Diastolic pressure, mmHg	70[63.8;80]	70[63.8;80]	70[60;70]	67.5[60;75]	3.334	0.343 ^b^
Height, cm	159.5[155.5;161]	157[153.5;162.3]	153[147.8;163.8]	160[158;166]	8.961	0.030 ^b,e,g^
Weight, kg	71.9 ± 21.5	67.2 ± 14.2	69.7 ± 13.1	70.7 ± 19.1	0.274	0.844 ^a^
Corrected weight ^†^, kg	58[54.8;68.5]	57[48.6;67.7]	60.8[52.3;74.8]	56.8[52;81]	0.694	0.875 ^b^
BMI (postpartum), kg/m^2^	26[24.1;32.4]	25.7[23.1;30.9]	29.4[25.7;34.3]	26.3[23.4;34.3]	2.635	0.451 ^b^
Corrected BMI ^†^, kg/cm^2^	26[24.1;32.4]	25.6[23.1;30.9]	29.4[25.7;34.3]	26.3[23.4;34.3]	2.748	0.432 ^b^
Fat mass, %	27.8[27.4;34.8]	28.5[24.3;34.3]	26.7[21.4;35.7]	27.7[23;35.7]	0.292	0.962 ^b^
Lean mass, %	72.2[65.2;73]	71.5[65.7;75.8]	73.4[64.4;78.6]	72.3[64.3;77]	1.274	0.735 ^b^
Total body water, %	49.6 ± 11.6	51.8 ± 7.9	51.4 ± 6.2	48.8 ± 8.8	0.480	0.697 ^a^
Phase angle	7.12 ± 2.77	5.90 ± 1.57	6.64 ± 2.88	7.40 ± 3.46	1.298	0.282 ^a^
Newborn’s temperature, °C	36.9[36.8;37]	36.7[36.4;37]	36.9[36.7;37]	36.9[36.8;37.1]	3.342	0.342 ^b^
NB length, cm	49.7 ± 2.0	49.7 ± 1.8	48.3 ± 3.5	48.9 ± 1.8	1.627	0.190 ^a^
NB weight, kg	3.30 ± 0.30	3.29 ± 0.49	3.04 ± 0.43	3.09 ± 0.38	2.058	0.113 ^a^
NB phase angle	5.2[4.15;9.00]	4.40[3.38;4.94]	4.80[3.76;6.22]	4.40[3.64;5.96]	1.010	0.799 ^b^
Mode of birth, n (%)					3.008	0.390 ^c^
Vaginal	9 (64.3)	16 (66.7)	7 (41.2)	11(61.1)		
C-section	5 (35.7)	8 (33.3)	10 (58.8)	7 (38.9)		
NB sex, n (%)					3.939	0.268 ^c^
Male	8 (53.3)	15 (65.2)	10 (52.6)	7 (35.0)		
Female	7 (46.7)	8 (34.8)	9 (47.4)	13 (65.0)		
Antibiotics *, n (%)					4.291	0.232 ^c^
Yes	11 (68.8)	12 (48.0)	8 (44.4)	10 (47.6)		
No	5 (31.3)	16 (64.0)	10 (55.6)	11 (52.4)		

Data are presented as median [IQR], mean ± standard deviation, or n (percentage). NNS: non-nutritive sweeteners; NB: newborn; t/wk: times per week; BMI: body mass index. ^†^ Corrected weight was calculated by subtracting 8 kg from postpartum weight. Corrected BMI was calculated using corrected weight. * Use of antibiotics at any point during the previous six months. ^a^ One-Way ANOVA; ^b^ Kruskal–Wallis: ^c^ χ^2^; ^d^ Q1 vs. Q4, *p* < 0.05 (Mann–Whitney U); ^e^ Q2 vs. Q3, *p* < 0.05 (Mann–Whitney U); ^f^ Q2 vs. Q4, *p* < 0.05 (Mann–Whitney U); ^g^ Q3 vs. Q4, *p* < 0.05 (Mann–Whitney U).

**Table 2:** Characteristics of the women’s daily diet included in the study. The dietary characteristics of participants are presented for each of the quartile groups of frequency of NNS consumption.

Variable	Q1 <4 t/wk *n* = 16	Q2 4 to <8 t/wk *n* = 25	Q3 8 to <16.5 t/wk) *n* = 20	Q4 ≥16.5 t/wk *n* = 21	H/χ^2^	*p*
Energy, kcal	1192[580;1475]	1256[941;1559]	1193[957;1673]	944.8[685;1640]	3.268 ^a^	0.352 ^a^
Lipids, g	38.7[14.3;46.3]	53.1[31.1;72.6]	42.9[36.1;68.9]	33.2[15.2;71.1]	4.911 ^a^	0.178 ^a^
Proteins, g	67.8[29.3;79.1]	59.2[42.5;79.7]	65[51.4;89.3]	51[29.4;65.1]	4.782 ^a^	0.188 ^a,f^
Protein Def, n (%)	9(56.3)	17(68.0)	14(70.0)	19(90.5)	5.757 ^b^	0.006 ^b^
HCO, g	151.7[82.4;196.5]	147.5[101.7;173.6]	130.1[92.9;190.7]	112.2[92.1;208.6]	1.691 ^a^	0.639 ^a^
HCO Def, n (%)	11(68.8)	14(56.0)	12(60.0)	14(66.7)	0.902 ^b^	0.821 ^b^
Fiber, g	10.7[4.1;21]	7.6[4;15.8]	6.2[3;14.7]	10.1[7.4;13.6]	4.480 ^a^	0.214 ^a^
Iron, mg	6.8[4.8;10.1]	7.4[4.3;10]	7.9[5.9;15.2]	5.3[4.7;11.8]	3.603 ^a^	0.308 ^a^
Iron Def, n (%)	16(100)	25(100)	20(100)	21(100)	---	---
Sodium, mg	788.3[183.2;1424.2]	1083.4[694.6;1804.4]	771.1[579.8;1952.7]	506.9[138;1320]	2.763 ^a^	0.430 ^a^
Na Def, n (%)	13(81.3)	16(64.0)	15(75.0)	18(85.7)	3.270 ^b^	0.352 ^b^
Potassium, mg	609.3[277.4;1130.2]	286.3[55.8;1082]	294.5[67.4;458.4]	701.5[363.9;961.7]	8.303 ^a^	0.040 ^a,f,f^
K Def, n (%)	15(93.8)	24(96.0)	20(100)	21(100)	2.258 ^b^	0.521 ^b^
Calcium, mg	493.8[18.1;637.5]	689.1[356.6;945.6]	567.7[460.1;1007.1]	373.5[120.1;571.1]	7.391 ^a^	0.060 ^a,e,f^
Ca Def, n (%)	15(93.8)	21(84.0)	16(80.0)	21(100)	5.268 ^b^	0.153 ^b^
Phosphorus, mg	0[0;0]	0[0;116.5]	0[0;0]	0[0;93.2]	6.630 ^a^	0.085 ^a,f^
P Def, n (%)	16(100)	25(100)	20(100)	21(100)	---	---
Sugar, g	32.1[17.3;62.3]	17.2[7.5;39.1]	7.9[0;18.5]	17.6[8;63.9]	11.234 ^a^	0.011 ^a,d,f^
Vitamin A, µg	232.3[105;553.2]	290.9[153.7;433.8]	251.3[166.6;432.2]	144.8[44.4;412]	3.070 ^a^	0.381 ^a^
Vit A Def, n (%)	14(87.5)	24(96.0)	17(85.0)	20(95.2)	2.434 ^b^	0.487 ^b^
Vitamin B9, µg	111.1[23.1;214.4]	74.7[17.2;153.7]	72.2[29.6;186.2]	58.8[23.1;157.3]	2.996 ^a^	0.392 ^a^
Vit B9 Def, n (%)	15(93.8)	24(96.0)	18(90.0)	21(100)	2.314 ^b^	0.510 ^b^
Vitamin C, mg	25.5[14.4;143.4]	14.3[1.1;149.7]	20.7[3;65.4]	24.9[9.6;75]	3.081 ^a^	0.379 ^a^
Vit CDef, n (%)	10(62.5)	18(72.0)	16(80.0)	15(71.4)	1.353 ^b^	0.717 ^b^
Selenium, µg	32[24.4;55.6]	24.6[11.8;33.3]	27.1[19.6;57.1]	23.1[1.5;46.6]	4.057 ^a^	0.255 ^a^
Sel Def, n (%)	16(100)	25(100)	20(100)	21(100)	---	---
Cholesterol, mg	120.5[28.8;281.4]	142.8[53.6;339.4]	192.5[142.4;309.3]	87.6[42.1;154.3]	6.277 ^a^	0.099 ^a,f^
Excess Chol, n (%)	3(18.8)	5(20.0)	4(20.0)	3(14.3)	0.316	0.957 ^b^
SFA, g	0.7[0;0.8]	2.3[0;6.3]	1[0;1.5]	0[0;5.1]	7.438 ^a^	0.059 ^a,c,e^
MFA, g	1.1[0;5.8]	5.8[0;12]	4.4[0;5.8]	0[0;9.3]	3.752 ^a^	0.289 ^a^
PFA, g	0.4[0;3]	3[0;4.6]	1.4[0;2.8]	0[0;2.6]	3.808 ^a^	0.283 ^a^
GI	437.1[213;538.8]	378.5[133;420.8]	253.8[118.6;375.7]	284[246;747.5]	3.653 ^a^	0.302 ^a^
GL	87[40.5;184.2]	90.9[32.9;94]	69.9[29.8;93.3]	80.4[66.7;148]	2.563 ^a^	0.463 ^a^

Data are presented as median [IQR] or n (percentage). t/wk: times per week; Def: deficiency (daily intake lower than recommended [45]); Vit: vitamin; Na: sodium; K: potassium; Ca: calcium; P: phosphorus; Sel: selenium; Kcal: kilocalories; Choo: cholesterol; SFA: saturated fatty acids; MFA: monounsaturated fatty acids; PFA: polyunsaturated fatty acids; GI; glycemic index; GL: glycemic load. ^a^ Kruskal–Wallis; ^b^ χ^2^; ^c^ Q1 vs. Q2, *p* < 0.05 (Mann–Whitney U); ^d^ Q1 vs. Q3, *p* < 0.05 (Mann–Whitney U); ^e^ Q2 vs. Q4, *p* < 0.05 (Mann–Whitney U); ^f^ Q3 vs. Q4, *p* < 0.05 (Mann–Whitney U).

## Data Availability

The data presented in this study are openly available in Zenodo at 10.5281/zenodo.10015589 and https://www.ncbi.nlm.nih.gov/sra/PRJNA1029649.

## Supplementary Materials

The following supporting information can be downloaded at: https://www.mdpi.com/article/10.3390/nu15234928/s1, Figure S1: Map of the Mexican Republic (latitude 23.634501, longitude −102.552784) that depicts the states from where the patients included in this study came from; Figure S2: Map of Mexico City municipalities from where the women included in the study came from (latitude 19.42847, longitude −99.12766); Figure S3: Optimal clusters. Optimal Number of clusters based on average silhouette width for (A) Weighted UniFrac and (B) Unweighted UniFrac. The Y-axis shows the average silhouette width; the x-axis indicates the number of clusters k. The blue vertical dashed line represents the max value of silhouette width at which the optimal number of clusters was determined; Figure S4: ANOSIM. Violin plots summarizing the ANOSIM model to test dissimilarities among groups and clusters. (A) Weighted UniFrac for NNS group comparisons, (B) Unweighted UniFrac for NNS group comparisons, (C) Weighted UniFrac for optimal clusters comparisons, (D) Unweighted UniFrac for NNS group comparisons; Figure S5: Distribution of the patients clustered in the NMDS scatter plot representing Unweighted UniFrac distance metrics in the different municipalities of Mexico City; Figure S6: Distribution of the patients clustered in the NMDS scatter plot representing Weighted UniFrac distance metrics in the different municipalities of Mexico City; Figure S7: Use of antibiotics. Bar plots comparing the number of pregnant women that used antibiotics within the 6 months previous to delivery in (A) the four groups of frequency of NNS consumption; (B) The optimal clusters obtained from Unweighted UniFrac; (C) The optimal clusters obtained from Weighted UniFrac; Figure S8: Bifidobacterium. Relative abundance boxplot of Bifidobacterium. Y-axis indicates relative abundance, x-axis shows NNS groups. The bracket enclosing Q1 and Q3 highlights statistical differences, according to Kruskal–Wallis; Tables S1-1 to S1-5: Analysis of qualitative and quantitative variables in relation to the Weighted and Unweighted clusters; Table S2: Alpha summary. Alpha diversity indexes for each group of frequency of NNS consumption. KW: Kruskal–Wallis; AOV: Analysis of Variance; Table S3: Kingdom statistics of the comparison between the quartiles of frequency of consumption of products with NNS with Kruskal–Wallis; Table S4: Phylum statistics of the comparison between the quartiles of frequency of consumption of products with NNS with Kruskal–Wallis; Table S5: Genus statistics of the comparison between the quartiles of frequency of consumption of products with NNS with Kruskal–Wallis; Table S6: Microbiome data analysis using DESeq2 to compare the quartiles of frequency of consumption of products with NNS.
