# Supplementary material for: Maternal Consumption of Non-Nutritive Sweeteners during Pregnancy Is Associated with Alterations in the Colostrum Microbiota
Source: Nutrients. 2023 Nov 26;15(23):4928. doi: 10.3390/nu15234928 (PMC10708104; doi:10.3390/nu15234928)
Supplement: Supplementary file 1 [file nutrients-15-04928-s001.zip › Supplementary figures 17oct.pdf]

nutrients

Maternal consumption of non-nutritive sweeteners during pregnancy is associated with alterations in the colostrum microbiota

Alejandro Tapia-González[1,†], Juan Manuel Vélez-Ixta[2,†], Nallely Bueno-Hernández[1], Alberto Piña-Escobedo[2], Jesús Carlos Briones-Garduño[3], Leticia de la Rosa-Ruiz[4], José Aguayo-Guerrero[1], Viridiana M. Mendoza-Martínez[1], Lenin Snowball-del-Pilar[1], Galileo Escobedo[1], Guillermo Meléndez-Mier[5], Lucía A. Méndez-García[1], Jaime García-Mena[2,*] and Marcela Esquivel-Velázquez[1,*]

[1] Laboratorio de Proteómica e Inmunometabolismo, Hospital General de México "Dr. Eduardo Liceaga". A.T.-G: alejandro_tapia@ciencias.unam.mx, N.B.-H.: nallely_bh5@yahoo.com, J.A.-G.: jose.aguayo01@iest.edu.mx, V.M.M.-M.: viridiana_2909@hotmail.com, L.S.-P.: benitojuarezgarciam@hotmail.com, G.E.: gescobedog@msn.com, L.A.M.-G.: lucia.angelica86@gmail.com.

[2] Departamento de Genética y Biología Molecular, Centro de Investigación y de Estudios Avanzados del Instituto Politécnico Nacional. A.P.-E.: apinae@cinvestav.mx, J.M.V.-I.: juan.velez@cinvestav.mx.

[3] Servicio de Ginecología, Hospital General de México "Dr. Eduardo Liceaga". drcarlosbriones@yahoo.com.mx

[4] Banco de Leche Humana y Lactancia, Hospital General de México "Dr. Eduardo Liceaga". letyfrose@gmail.com

[5] Facultad de Salud Pública y Nutrición, Universidad Autónoma de Monterrey. melendez651@gmail.com

† These authors contributed equally to this work.

* Correspondence: M.E.-V.: esquivel.marcela@gmail.com; J.G.-M.: jgmena@cinvestav.mx; Tel.: M.E.-V.: +52 55 2789 2000 Ext. 5652 or 5658; J.G.-M.: +52 55 5747 3800 Ext. 5327.

Supplementary figures

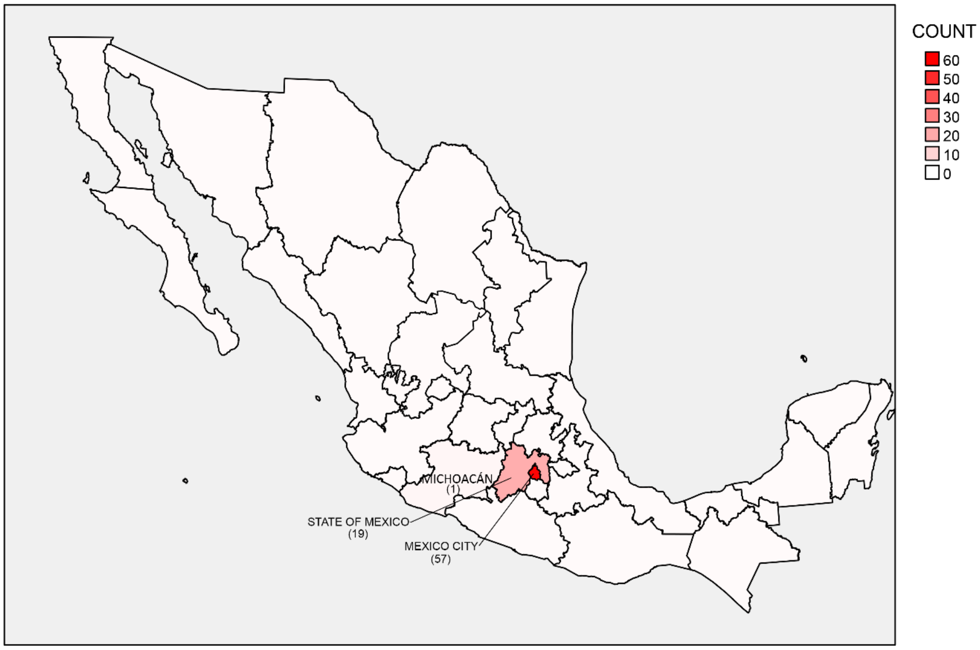

Figure S1. Map of the Mexican Republic (latitude 23.634501, longitude -102.552784) that depicts the states from where the patients included in this study came from.

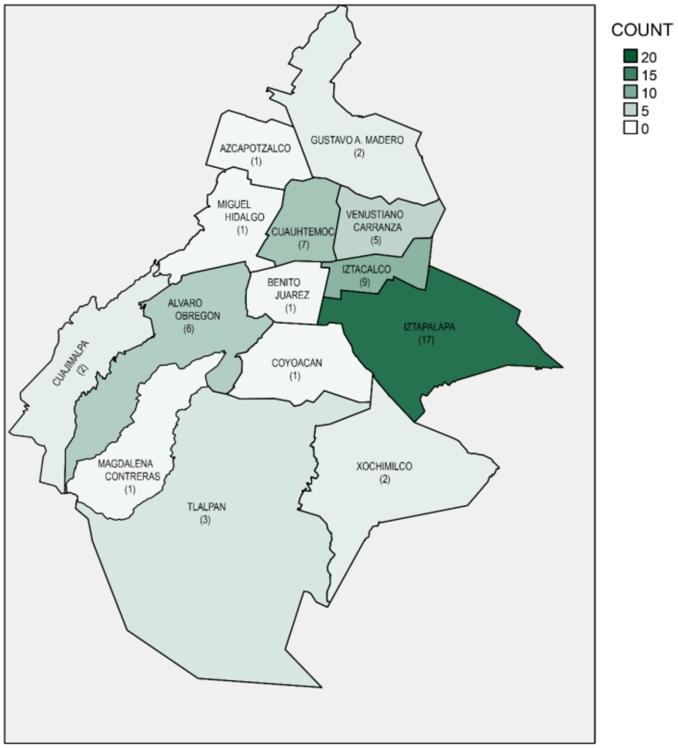

Figure S2. Map of Mexico City municipalities from where the women included in the study came from (latitude 19.42847, longitude 99.12766).

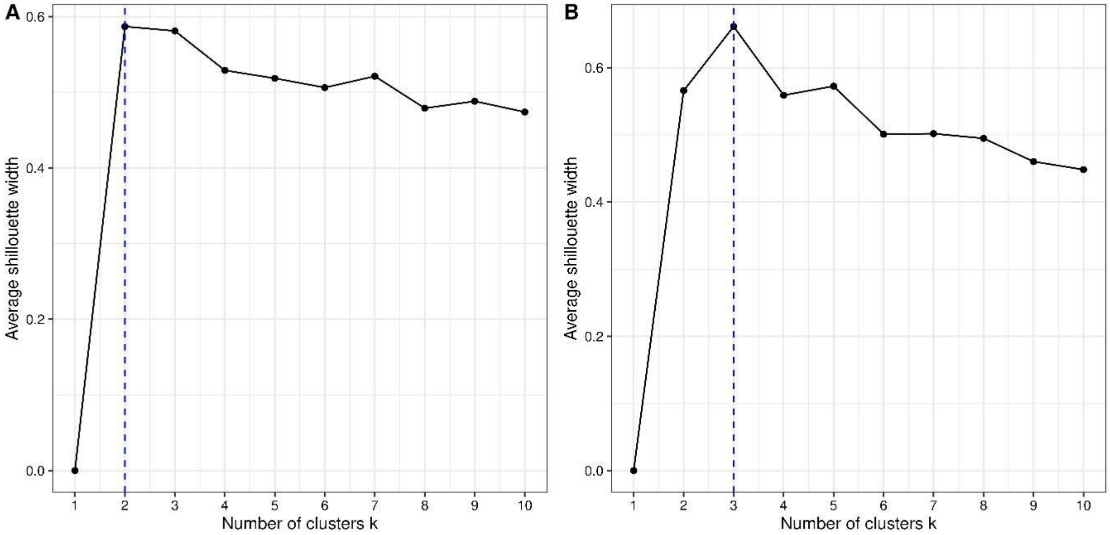

Figure S3. Optimal clusters. Optimal Number of clusters based on average silhouette width for **A)** Weighted UniFrac and **B)** Unweighted UniFrac. The Y-axis shows the average silhouette width; the x-axis indicates the number of clusters k. The blue vertical dashed line represents the max value of silhouette width at which the optimal number of clusters was determined.

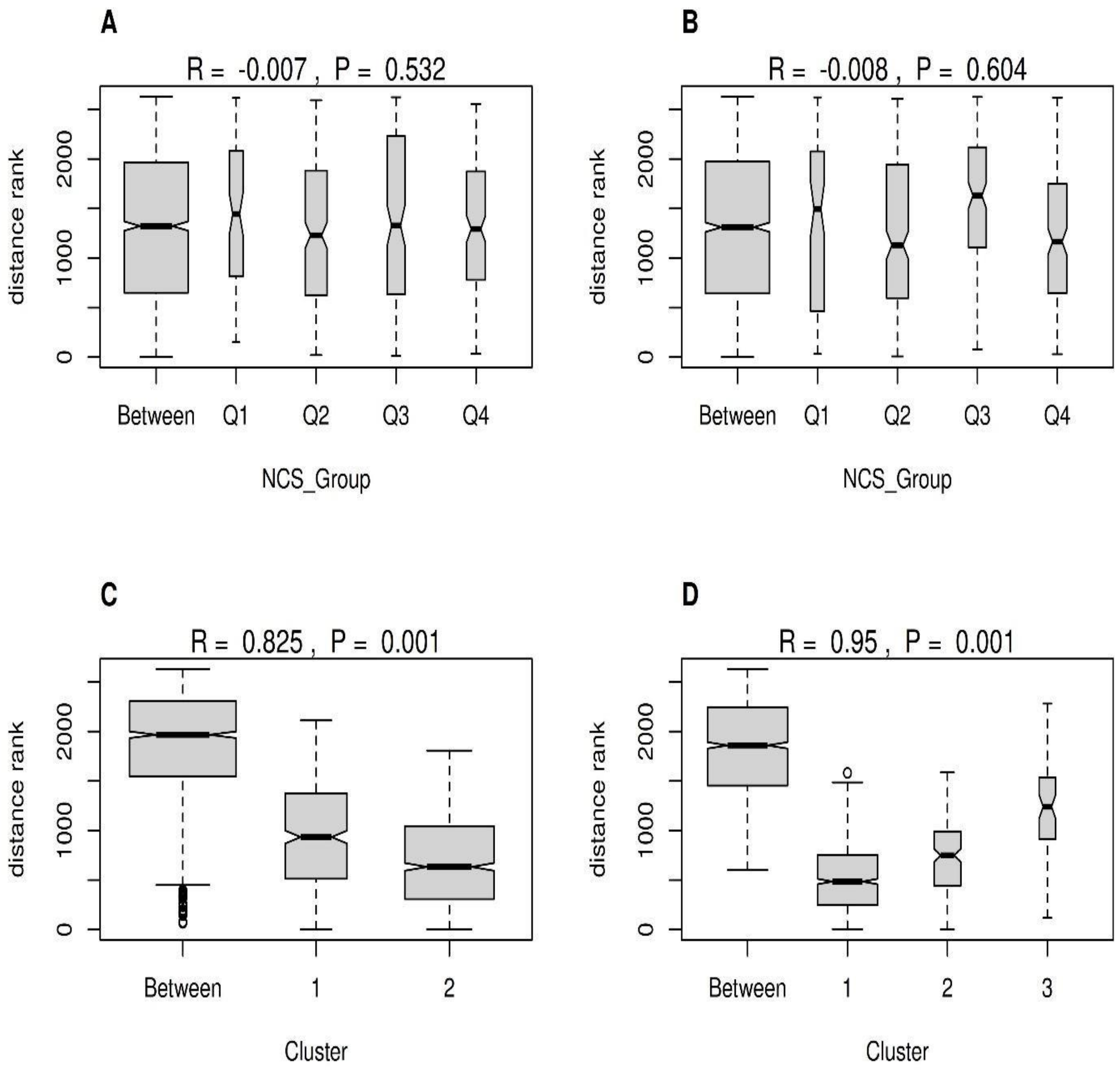

Figure S4. ANOSIM. Violin plots summarizing ANOSIM model to test dissimilarities among groups and clusters. A) Weighted UniFrac for NNS group comparisons, B) Unweighted UniFrac for NNS group comparisons, C) Weighted UniFrac for optimal clusters comparisons, D) Unweighted UniFrac for NCS group comparisons.

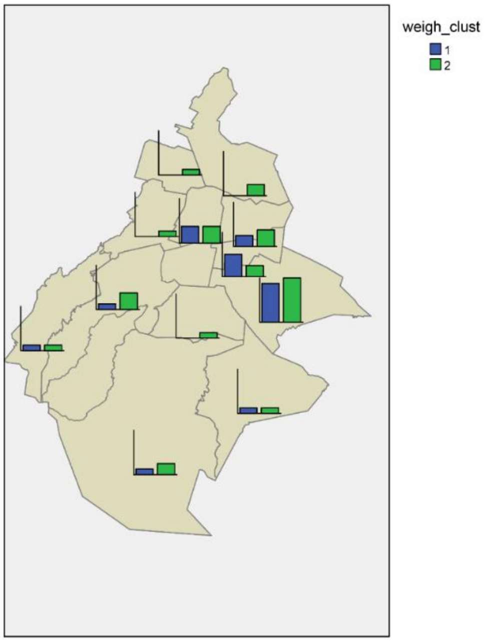

Figure S5. Distribution of the patients clustered in the NMDS scatter plot representing Unweighted UniFrac distance metrics in the different municipalities of Mexico City.

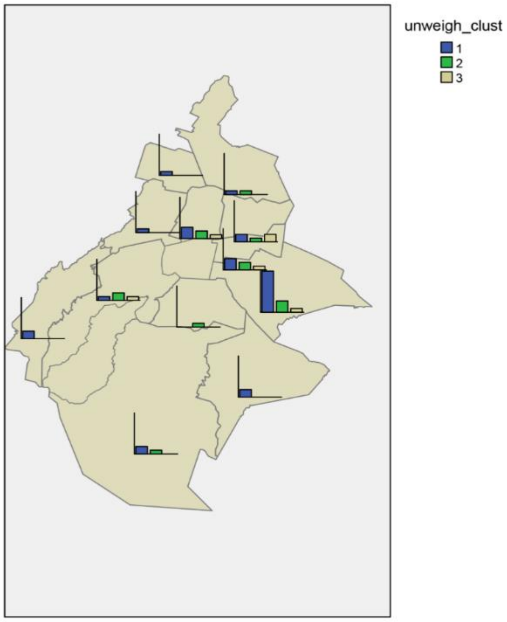

Figure S6. Distribution of the patients clustered in the NMDS scatter plot representing Weighted UniFrac distance metrics in the different municipalities of Mexico City.

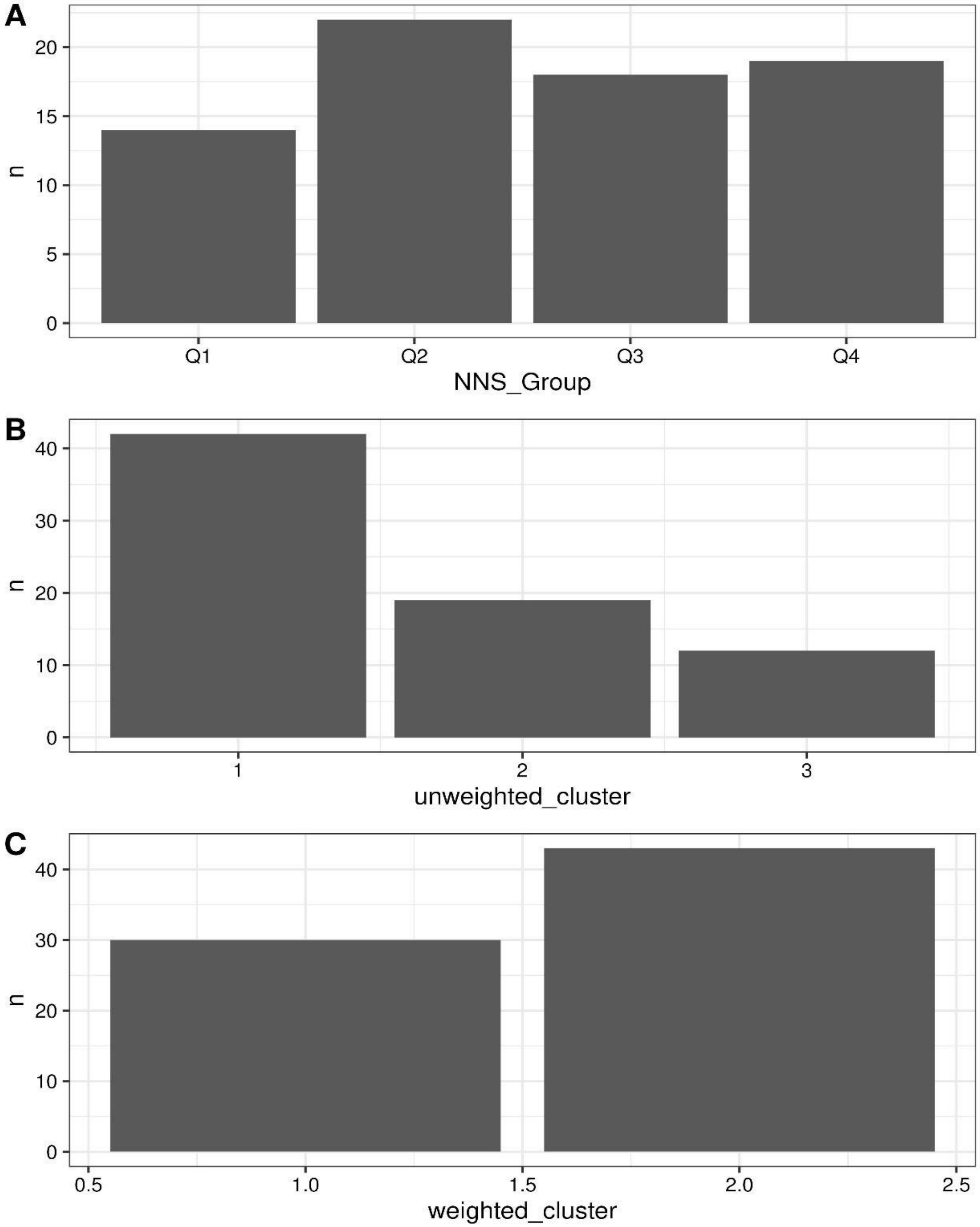

Figure S7. Use of antibiotics. Bar plots comparing the number of pregnant women that used antibiotics within the 6 months previous to delivery in A) the four groups of frequency of NNS consumption; B) The optimal clusters obtained from Unweighted UniFrac; C) The optimal clusters obtained from Weighted UniFrac.

nutrients MDPI

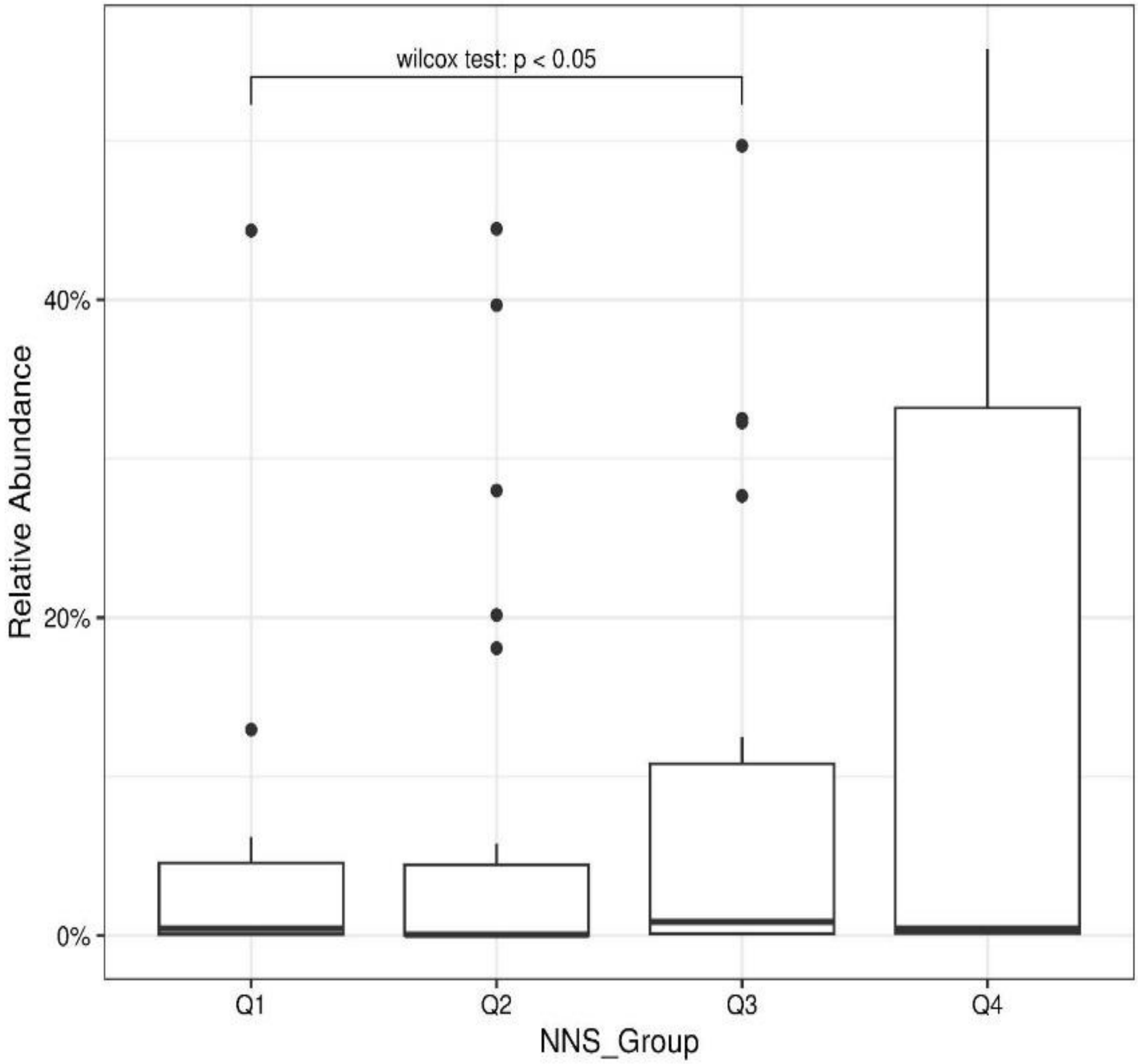

Figure S8. Bifidobacterium. Relative abundance boxplot of Bifidobacterium. Y-axis indicates relative abundance, x-axis shows NNS groups. The bracket enclosing Q1 and Q3 highlights statistical differences, according to Kruskal-Wallis.
