# Supplementary material for: Maternal Consumption of Non-Nutritive Sweeteners during Pregnancy Is Associated with Alterations in the Colostrum Microbiota
Source: Nutrients. 2023 Nov 26;15(23):4928. doi: 10.3390/nu15234928 (PMC10708104; doi:10.3390/nu15234928)
Supplement: Supplementary file 1 [file nutrients-15-04928-s001.zip › SUpplementary tables final 17oct.pdf]

nutrients

MDPI

Maternal consumption of non-nutritive sweeteners during pregnancy is associated with alterations in the colostrum microbiota.

Alejandro Tapia-González[1,†], Juan Manuel Vélez-Ixta[2,†], Nallely Bueno-Hernández[1], Alberto Piña-Escobedo[2], Jesús Carlos Briones-Garduño[3], Leticia de la Rosa-Ruiz[4], José Aguayo-Guerrero[1], Viridiana M. Mendoza-Martínez[1], Lenin Snowball-del-Pilar[1], Galileo Escobedo[1], Guillermo Meléndez-Mier[5], Lucía A. Méndez-García[1], Jaime García-Mena[2,*] and Marcela Esquivel-Velázquez[1,*]

[1] Laboratorio de Proteómica e Inmunometabolismo, Hospital General de México "Dr. Eduardo Liceaga". A.T.-G: alejandro_tapia@ciencias.unam.mx, N.B.-H.: nallely_bh5@yahoo.com, J.A.-G.: jose.aguayo01@iest.edu.mx, V.M.M.-M.: viridiana_2909@hotmail.com, L.S.-P.: benitojuarezgarciam@hotmail.com, G.E.: gescobedog@msn.com, L.A.M.-G.: lucia.angelica86@gmail.com.

[2] Departamento de Genética y Biología Molecular, Centro de Investigación y de Estudios Avanzados del Instituto Politécnico Nacional. A.P.-E.: apinae@cinvestav.mx, J.M.V.-I.: juan.velez@cinvestav.mx.

[3] Servicio de Ginecología, Hospital General de México "Dr. Eduardo Liceaga". drcarlosbriones@yahoo.com.mx

[4] Banco de Leche Humana y Lactancia, Hospital General de México "Dr. Eduardo Liceaga". letyfrose@gmail.com

[5] Facultad de Salud Pública y Nutrición, Universidad Autónoma de Monterrey. melendez651@gmail.com

† These authors contributed equally to this work.

* Correspondence: M.E.-V.: esquivel.marcela@gmail.com; J.G.-M.: jgmena@cinvestav.mx; Tel.: M.E.-V.: +52 55 2789 2000 Ext. 5652 or 5658; J.G.-M.: +52 55 5747 3800 Ext. 5327.

Supplementary tables

Table S1. Analysis of qualitative and quantitative variables in relation to the Weighted and Unweighted clusters.

		Weighted clusters					*Unweighted clusters*			
		1	2	3	χ^2/t	*p*	1	2	χ^2/F	*p*
CDMX	YES	29(70.7)	13(81.3)	6(60)	1.411	0.494[a]	20(74.1)	28(70)	.132	0.717[a]
	NO	12(29.3)	3(18.8)	4(40)			7(25.9)	12(30)		
ZMVM	YES	40(97.6)	15(93.8)	9(90)	1.229	0.541[a]	27(100)	37(92.5)	2.120	0.145[a]
	NO	1(2.4)	1(6.3)	1(10)			0(0)	3(7.5)		
Father DBT	YES	4(10.3)	2(11.1)	6(54.5)	12.300	0.002[a]	4(14.3)	8(20)	.370	0.543[a]
	NO	35(89.7)	16(88.9)	5(45.5)			24(85.7)	32(80)		
Father HTN	YES	2(5.1)	1(5.6)	2(18.2)	2.262	0.323[a]	1(3.6)	4(10)	.999	0.318[a]
	NO	37(94.9)	17(94.4)	9(81.8)			27(96.4)	36(90)		
Father TB	YES	0(0)	0(0)	0(0)	----	----	0(0)	0(0)	----	----
	NO	39(100)	18(100)	11(100)			28(100)	40(100)		
Father OB	YES	2(5.1)	1(5.6)	2(18.2)	2.262	0.323[a]	2(7.1)	3(7.5)	.003	0.956[a]
	NO	37(94.9)	17(94.4)	9(81.8)			26(92.9)	37(92.5)		
Father MS	YES	1(2.6)	1(5.6)	0(0)	0.784	0.676[a]	1(3.6)	1(2.5)	.066	0.797[a]

	NO	38(97.4)	17(94.4)	11(100)			27(96.4)	39(97.5)		
Mother DBT	YES	5(11.9)	1(5.6)	3(25)	2.522	0.283[a]	2(6.7)	7(16.7)	1.600	0.206[a]
	NO	37(88.1)	17(94.4)	9(75)			28(93.3)	35(83.3)		
Mother HTN	YES	5(11.9)	3(16.7)	4(33.3)	3.086	0.214[a]	4(13.3)	8(19)	.411	0.521[a]
	NO	37(88.1)	15(83.3)	8(66.7)			26(86.7)	34(81)		
Mother TB	YES	1(2.4)	1(5.6)	0(0)	0.882	0.644[a]	0(0)	2(4.8)	1.469	0.225[a]
	NO	41(97.6)	17(94.4)	12(100)			30(100)	40(95.2)		
Mother OB	YES	1(2.4)	1(5.6)	1(8.3)	0.944	0.624[a]	1(3.3)	2(4.8)	.089	0.765[a]
	NO	41(97.6)	17(94.4)	11(91.7)			29(96.7)	40(95.2)		
Mother MS	YES	1(2.4)	1(5.6)	1(8.3)	0.944	0.624[a]	1(3.3)	2(4.8)	.089	0.765[a]
	NO	41(97.6)	17(94.4)	11(91.7)			29(96.7)	40(95.2)		
Brother DBT	YES	4(9.5)	0(0)	1(8.3)	1.812	0.404[a]	1(3.3)	4(9.5)	1.038	0.308[a]
	NO	38(90.5)	18(100)	11(91.7)			29(96.7)	38(90.5)		
Brother HTN	YES	2(4.8)	0(0)	0(0)	1.469	0.480[a]	0(0)	2(4.8)	1.469	0.225[a]
	NO	40(95.2)	18(100)	12(100)			30(100)	40(95.2)		
Brother TB	YES	1(2.4)	0(0)	1(8.3)	1.910	0.385[a]	0(0)	2(4.8)	1.469	0.225[a]
	NO	41(97.6)	18(100)	11(91.7)			30(100)	40(95.2)		
Brother OB	YES	1(2.4)	1(5.6)	0(0)	0.882	0.644[a]	0(0)	2(4.8)	1.469	0.225[a]
	NO	41(97.6)	17(94.4)	12(100)			30(100)	40(95.2)		
Brother MS	YES	1(2.4)	0(0)	0(0)	0.724	0.696[a]	0(0)	1(2.4)	.725	0.395[a]
	NO	41(97.6)	18(100)	12(100)			30(100)	41(97.6)		
Alcohol	YES	0(0)	0(0)	0(0)	8.471	0.076[a]	0(0)	0(0)	2.261	0.323[a]
	wkends	0(0)	0(0)	1(8.3)			0(0)	1(2.4)		
	Occas	6(14.3)	2(11.1)	4(33.3)			7(23.3)	5(11.9)		
	Never	36(85.7)	16(88.9)	7(58.3)			23(76.7)	36(85.7)		
Tabacco	YES	14(33.3)	5(27.8)	5(41.7)	5.429	0.246[a]	9(30)	15(35.7)	.862	0.350[a]
	Occas	3(7.1)	1(5.6)	3(25)			4(13.3)	3(7.1)		
	Never	25(59.5)	12(66.7)	4(33.3)			17(56.7)	24(57.1)		
Drugs	YES	0(0)	0(0)	0(0)	----	----	0(0)	0(0)	----	----
	NO	42(100)	18(100)	12(100)			30(100)	42(100)		
Cancer	YES	0(0)	0(0)	0(0)	----	----	0(0)	0(0)	----	----
	NO	42(100)	18(100)	12(100)			30(100)	42(100)		
Alergies	1	8(0)	1(0)	1(0)	2.128	0.345[a]	4(0)	6(0)	.024	0.876[a]
	2	34(0)	16(0)	11(0)			26(0)	35(0)		
NB Sex	Male	14(35)	11(73.3)	6(50)	6.530	0.380[a]	11(39.3)	20(51.3)	.943	0.331[a]
	Female	26(65)	4(26.7)	6(50)			17(60.7)	19(48.7)		
Antibiotic use	YES	22(53.7)	7(38.9)	3(27.3)	2.888	0.236[a]	13(44.8)	19(46.3)	.016	0.900[a]
	NO	19(46.3)	11(61.1)	8(72.7)			16(55.2)	22(53.7)		
Antibiotic use in the last 6 months	YES	22(53.7)	8(44.4)	4(36.4)	1.204	0.548[a]	9(31)	25(61)	6.096	0.017[a]
	NO	19(46.3)	10(55.6)	7(63.6)			20(69)	16(39)		
Other medication	YES	31(73.8)	9(52.9)	6(54.5)	3.062	0.216[a]	21(70)	25(62.5)	.428	0.513[a]
	NO	11(26.2)	8(47.1)	5(45.5)			9(30)	15(37.5)		
BMI	underweigth	0(0)	0(0)	1(9.1)	6.119	0.410[a]	1(3.6)	0(0)	2.842	0.417[a]
	normal	11(27.5)	5(27.8)	4(36.4)			10(35.7)	10(24.4)		
	overweigth	17(42.5)	7(38.9)	3(27.3)			9(32.1)	18(43.9)		

	obesity	12(30)	6(33.3)	3(27.3)			8(28.6)	13(31.7)		
NNS consumption	NO	21(50)	6(33.3)	4(33.3)	1.983	0.371[a]	16(53.3)	15(35.7)	2.216	0.137[a]
	YES	21(50)	12(66.7)	8(66.7)			14(46.7)	27(64.3)		
milk	NO	1(2.4)	0(0)	0(0)	0.724	0.696[a]	0(0)	1(2.4)	.724	0.395[a]
	YES	41(97.6)	18(100)	12(100)			30(100)	41(97.6)		
yogurt	YES	40(95.2)	17(94.4)	10(83.3)	2.119	0.347[a]	29(96.7)	38(90.5)	1.038	0.308[a]
	NO	2(4.8)	1(5.6)	2(16.7)			1(3.3)	4(9.5)		
Chocolate	YES	32(76.2)	14(77.8)	9(75)	0.033	0.984[a]	22(73.3)	33(78.6)	.266	0.606[a]
	NO	10(23.8)	4(22.2)	3(25)			8(26.7)	9(21.4)		
Popped cereals	YES	35(83.3)	14(77.8)	11(91.7)	1.000	0.607[a]	24(80)	36(85.7)	.411	0.521[a]
	NO	7(16.7)	4(22.2)	1(8.3)			6(20)	6(14.3)		
Coockies Maria style	YES	28(66.7)	10(55.6)	7(58.3)	0.770	0.680[a]	18(60)	27(64.3)	.137	0.711[a]
	NO	14(33.3)	8(44.4)	5(41.7)			12(40)	15(35.7)		
Choco-cream cockies	YES	29(69)	11(61.1)	8(66.7)	0.357	0.836[a]	19(63.3)	29(69)	.257	0.612[a]
	NO	13(31)	7(38.9)	4(33.3)			11(36.7)	13(31)		
muffines	YES	29(69)	11(61.1)	7(58.3)	0.657	0.720[a]	19(63.3)	28(66.7)	.086	0.770[a]
	NO	13(31)	7(38.9)	5(41.7)			11(36.7)	14(33.3)		
Donuts and croassants	YES	19(45.2)	6(33.3)	6(50)	1.012	0.603[a]	14(46.7)	17(40.5)	.274	0.601[a]
	NO	23(54.8)	12(66.7)	6(50)			16(53.3)	25(59.5)		
Salad	YES	36(85.7)	14(77.8)	12(100)	2.986	0.225[a]	22(73.3)	40(95.2)	7.021	0.008[a]
	NO	6(14.3)	4(22.2)	0(0)			8(26.7)	2(4.8)		
Spinach, swiss chard	YES	25(59.5)	8(44.4)	10(83.3)	4.528	0.104[a]	20(66.7)	23(54.8)	1.031	0.310[a]
	NO	17(40.5)	10(55.6)	2(16.7)			10(33.3)	19(45.2)		
Vegetables on the side	YES	28(66.7)	12(66.7)	10(83.3)	1.309	0.520[a]	22(73.3)	28(66.7)	.367	0.545[a]
	NO	14(33.3)	6(33.3)	2(16.7)			8(26.7)	14(33.3)		
Potatoes	YES	36(85.7)	15(83.3)	12(100)	2.122	0.346[a]	26(86.7)	37(88.1)	.033	0.857[a]
	NO	6(14.3)	3(16.7)	0(0)			4(13.3)	5(11.9)		
Legumes	YES	38(90.5)	16(88.9)	10(83.3)	0.482	0.786[a]	26(86.7)	38(90.5)	.257	0.612[a]
	NO	4(9.5)	2(11.1)	2(16.7)			4(13.3)	4(9.5)		
Rice	YES	38(90.5)	17(94.4)	11(91.7)	0.260	0.878[a]	29(96.7)	37(88.1)	1.683	0.195[a]
	NO	4(9.5)	1(5.6)	1(8.3)			1(3.3)	5(11.9)		
Pasta	YES	41(97.6)	17(94.4)	12(100)	0.882	0.644[a]	29(96.7)	41(97.6)	.059	0.808[a]
	NO	1(2.4)	1(5.6)	0(0)			1(3.3)	1(2.4)		
Creamy soups	YES	23(54.8)	12(66.7)	9(75)	1.920	0.383[a]	18(60)	26(61.9)	.027	0.870[a]
	NO	19(45.2)	6(33.3)	3(25)			12(40)	16(38.1)		
Eggs	YES	37(88.1)	16(88.9)	11(91.7)	0.121	0.642[a]	27(90)	37(88.1)	.064	0.800[a]
	NO	5(11.9)	2(11.1)	1(8.3)			3(10)	5(11.9)		
Chicken, turkey	YES	41(97.6)	16(88.9)	12(100)	3.031	0.220[a]	29(96.7)	40(95.2)	.089	0.765[a]
	NO	1(2.4)	2(11.1)	0(0)			1(3.3)	2(4.8)		
Beef, pig, lamb	YES	39(92.9)	17(94.4)	10(83.3)	1.351	0.509[a]	29(96.7)	37(88.1)	1.683	0.195[a]
	NO	3(7.1)	1(5.6)	2(16.7)			1(3.3)	5(11.9)		
Minced meat	YES	29(69)	14(77.8)	8(66.7)	0.586	0.746[a]	20(66.7)	31(73.8)	.432	0.511[a]
	NO	13(31)	4(22.2)	4(33.3)			10(33.3)	11(26.2)		
White fish	YES	22(52.4)	8(44.4)	8(66.7)	1.433	0.488[a]	13(43.3)	25(59.5)	1.841	0.175[a]
	NO	20(47.6)	10(55.6)	4(33.3)			17(56.7)	17(40.5)		

Blue fish	YES	14(33.3)	8(44.4)	7(58.3)	2.598	0.273[a]	13(43.3)	16(38.1)	.200	0.655[a]
	NO	28(66.7)	10(55.6)	5(41.7)			17(56.7)	26(61.9)		
Seafood	YES	15(35.7)	7(38.9)	3(25)	0.657	0.720[a]	9(30)	16(38.1)	.506	0.477[a]
	NO	27(64.3)	11(61.1)	9(75)			21(70)	26(61.9)		
Croquettes	YES	23(54.8)	10(55.6)	9(75)	1.649	0.438[a]	17(56.7)	25(59.5)	.059	0.808[a]
	NO	19(45.2)	8(44.4)	3(25)			13(43.3)	17(40.5)		
Berad	YES	24(57.1)	12(66.7)	9(75)	1.448	0.485[a]	23(76.7)	22(52.4)	4.404	0.036[a]
	NO	18(42.9)	6(33.3)	3(25)			7(23.3)	20(47.6)		
Ham	YES	38(90.5)	15(83.3)	10(83.3)	0.816	0.665[a]	27(90)	36(85.7)	.294	0.588[a]
	NO	4(9.5)	3(16.7)	2(16.7)			3(10)	6(14.3)		
White cheese	YES	36(85.7)	15(83.3)	11(91.7)	0.431	0.806[a]	26(86.7)	36(85.7)	.013	0.908[a]
	NO	6(14.3)	3(16.7)	1(8.3)			4(13.3)	6(14.3)		
Other cheese	YES	22(52.4)	6(33.3)	5(41.7)	1.942	0.379[a]	16(53.3)	17(40.5)	1.165	0.280[a]
	NO	20(47.6)	12(66.7)	7(58.3)			14(46.7)	25(59.5)		
Citric fruits	YES	40(95.2)	17(94.4)	12(100)	0.646	0.724[a]	29(96.7)	40(95.2)	.089	0.765[a]
	NO	2(4.8)	1(5.6)	0(0)			1(3.3)	2(4.8)		
Other fruits	YES	38(90.5)	17(94.4)	12(100)	1.382	0.501[a]	26(86.7)	41(97.6)	3.248	0.071[a]
	NO	4(9.5)	1(5.6)	0(0)			4(13.3)	1(2.4)		
Canned fruit	YES	15(35.7)	5(27.8)	4(33.3)	0.357	0.836[a]	11(36.7)	13(31)	.257	0.612[a]
	NO	27(64.3)	13(72.2)	8(66.7)			19(63.3)	29(69)		
Natural fruit juice	YES	31(73.8)	11(61.1)	11(91.7)	3.463	0.177[a]	23(76.7)	30(71.4)	.247	0.619[a]
	NO	11(26.2)	7(38.9)	1(8.3)			7(23.3)	12(28.6)		
Commercial fruit juice	YES	28(66.7)	14(77.8)	3(25)	9.304	0.010[a]	17(56.7)	28(66.7)	.747	0.388[a]
	NO	14(33.3)	4(22.2)	9(75)			13(43.3)	14(33.3)		
Nuts	YES	25(59.5)	11(61.1)	8(66.7)	0.200	0.905[a]	19(63.3)	25(59.5)	.107	0.744[a]
	NO	17(40.5)	7(38.9)	4(33.3)			11(36.7)	17(40.5)		
Dairy desserts	YES	22(52.4)	8(44.4)	6(50)	0.317	0.853[a]	17(56.7)	19(45.2)	.914	0.339[a]
	NO	20(47.6)	10(55.6)	6(50)			13(43.3)	23(54.8)		
Cakes	YES	13(31)	11(61.1)	5(41.7)	4.776	0.092[a]	11(36.7)	18(42.9)	.279	0.597[a]
	NO	29(69)	7(38.9)	7(58.3)			19(63.3)	24(57.1)		
Fry bags	YES	30(71.4)	14(77.8)	7(58.3)	1.335	0.513[a]	24(80)	27(64.3)	2.092	0.148[a]
	NO	12(28.6)	4(22.2)	5(41.7)			6(20)	15(35.7)		
Candies	YES	31(73.8)	12(66.7)	8(66.7)	0.432	0.806[a]	21(70)	30(71.4)	.017	0.895[a]
	NO	11(26.2)	6(33.3)	4(33.3)			9(30)	12(28.6)		
Ice cream	YES	29(69)	12(66.7)	10(83.3)	1.124	0.570[a]	20(66.7)	31(73.8)	.432	0.511[a]
	NO	13(31)	6(33.3)	2(16.7)			10(33.3)	11(26.2)		
Soda	YES	27(64.3)	16(88.9)	6(50)	5.668	0.059[a]	18(60)	31(73.8)	1.535	0.215[a]
	NO	15(35.7)	2(11.1)	6(50)			12(40)	11(26.2)		
Ligth soda	YES	2(4.8)	3(16.7)	1(8.3)	2.338	0.311[a]	4(13.3)	2(4.8)	.1683	0.195[a]
	NO	40(95.2)	15(83.3)	11(91.7)			26(86.7)	40(95.2)		
Wine	YES	2(4.8)	1(5.6)	2(16.7)	2.119	0.347[a]	3(10)	2(4.8)	.743	0.389[a]
	NO	40(95.2)	17(94.4)	10(83.3)			27(90)	40(95.2)		
Beer	YES	11(26.2)	3(16.7)	4(33.3)	1.143	0.565[a]	9(30)	9(21.4)	.686	0.408[a]
	NO	31(73.8)	15(83.3)	8(66.7)			21(70)	33(78.6)		
	YES	1(2.4)	0(0)	0(0)	0.724	0.696[a]	1(3.3)	0(0)	1.420	0.233[a]

Non-alcohol beer	NO	41(97.6)	18(100)	12(100)			29(96.7)	42(100)		
Distilled drinks	YES	3(7.1)	0(0)	2(16.7)	3.101	0.212[a]	3(10)	2(4.8)	.743	0.389[a]
	NO	39(92.9)	18(100)	10(83.3)			27(90)	40(95.2)		
Age, years		23.9±4.56	25.16±6.97	25.17±4.3	0.510	0.603[b]	24.1±5.12	24.67±5.33	-0.487	0.628[c]
Brothers, n		2.68±1.6	2.94±1.92	2.18±0.98	0.761	0.471[b]	2.5±1.59	2.8±1.64	-0.745	0.459[c]
Children, n		1.48±0.63	1.78±1.06	1.92±0.79	1.925	0.154[b]	1.67±0.84	1.6±0.77	0.295	0.769[c]
Gestational age (LMP/USG), weeks		38.82±1.77	38.88±1.95	39.05±1.5	0.076	0.927[b]	39.08±1.81	38.72±1.72	0.949	0.346[c]
Age of menarch		12.51±1.73	12.63±1.34	12.42±1.44	0.071	0.931[b]	12.53±1.8	12.52±1.42	0.186	0.853[c]
Age of sexual initiation, years		17.56±2.09	17.11±3.14	17.25±2.77	0.226	0.798[b]	17.37±2.53	17.41±2.47	-0.055	0.956[c]
Pregnancies, n		1.9±0.86	2.21±0.98	2.42±1.08	1.716	0.187[b]	1.97±1.07	2.14±0.84	-0.905	0.369[c]
Births, n		0.83±0.77	1.21±1.08	0.92±1.24	1.058	0.353[b]	0.97±1.13	0.93±0.81	0.173	0.864[c]
C-sections, n		0.68±0.85	0.58±0.84	1±1.21	0.819	0.445[b]	0.67±0.84	0.74±0.96	-0.405	0.687[c]
Abortions, n		0.36±0.53	0.26±0.45	0.25±0.62	0.315	0.731[b]	0.2±0.41	0.4±0.58	-1.648	0.104[c]
Gestational Age by Capurro, weeks		39.61±2.45	39.45±1.5	40.01±1.35	0.259	0.773[b]	39.53±1.71	39.72±2.33	-0.365	0.716[c]
Mothers temperature, °C		36.49±0.4	36.42±0.47	36.37±0.24	0.474	0.265[b]	36.39±0.39	36.5±0.4	-0.980	0.330[c]
Mother's systolic pressure, mmHg		104.8±11.2	106±10.2	102.91±7.82	0.302	0.740[b]	105.59±10.13	104.31±10.67	0.661	0.511[c]
Mother's diastolic pressure, mmHg		67.68±10.86	69.74±7.72	68.18±10.79	0.269	0.765[b]	70.17±8.18	67.02±11	1.325	0.190[c]
Mother's heigth, cm		1.58±0.07	1.59±0.07	1.6±0.09	0.346	0.709[b]	1.6±0.07	1.57±0.07	1.582	0.118[c]
Mothers weigth after delivery, kg		68.81±14.45	70.55±15.11	68.11±15.46	0.123	0.885[b]	69±15.55	69.29±14.11	-0.066	0.947[c]
Mother's lean mass, %		20.6±9.81	20.3±8.57	17.71±12.77	0.302	0.741[b]	20.88±11.15	19.49±8.94	0.529	0.599[c]
Mothers fat mass, %		27.76±7.65	30.15±10.09	23.29±14.55	1.482	0.236[b]	27.88±8.87	27.69±10.45	0.076	0.940[c]
Mothers lean mass, kg		50.7±9	45.35±7.02	51.53±8.84	2.608	0.083[b]	49.25±8.21	49.27±9.13	-0.009	0.993[c]
Mother's fat mass, kg		72.26±7.65	69.85±10.09	76.71±14.55	1.482	0.236[b]	72.15±8.88	72.31±10.45	-0.063	0.950[c]
Mothers total body water, kg		35.78±6.39	31.62±5.51	36.92±6.73	3.151	0.051[b]	34.83±6.03	34.67±6.79	0.096	0.924[c]
Mother's total body water, %		51.03±5.6	47.51±10.49	55±10.98	2.548	0.087[b]	51.02±6.49	50.32±9.64	0.309	0.758[c]
Neborn's temperature, °C		36.89±0.3	36.73±0.36	36.97±0.2	2.589	0.083[b]	36.89±0.21	36.84±0.37	0.694	0.490[c]
Newborn's length, cm		49.05±2.12	49.17±2.38	48.82±3.71	0.067	0.935[b]	49.14±2.18	48.98±2.65	0.269	0.789[c]
Newborn's weight, kg		3.17±0.45	3.2±0.39	3.21±0.52	0.043	0.958[b]	3.11±0.43	3.23±0.44	-1.053	0.296[c]
Mother's BMI		564.97±1242.72	1922.07±3584.44	166.61±221.81	0.200	0.819[b]	548.62±1261.81	1064.86±2559.29	-0.922	0.360[c]
NNS, mg/week		564.97±1242.72	1922.07±3584.44	166.61±221.81	3.659	0.031[b]	548.62±1261.81	1064.86±2559.29	-1.058	0.294[c]
Yogurts, times/week		1.55±2.29	1.26±1.37	2±2.41	0.448	0.641[b]	0.9±1.67	2±2.26	-2.161	0.034[c]
Jams, times/week		0.79±2.09	1.37±2.01	0.5±1.24	0.864	0.426[b]	0.83±2.23	0.93±1.76	-0.252	0.802[c]
Candies, times/week		3.4±4.38	2.95±3.61	2.67±3.45	0.191	0.826[b]	3.07±4.57	3.23±3.62	-0.251	0.803[c]
ASB, times/week		0.98±2.39	0.74±2.33	0.33±1.15	0.402	0.671[b]	0.93±2.49	0.72±2.02	0.365	0.716[c]
Cereals, times/week		0.81±2.06	0±0	0.5±1.73	1.452	0.241[b]	1.07±2.43	0.19±0.85	2.156	0.035[c]
Sweeteners, times/week		10.76±12.71	8.95±6.71	10.33±7.28	0.189	0.828[b]	9.53±13.78	10.7±7.77	-0.518	0.606[c]
NNS, times/week		12.6±12.57	14.79±11.18	12.08±11.2	0.268	0.766[b]	11.7±11.09	14.05±12.47	-0.906	0.368[c]
Energy, kcal		1298.61±598.67	1196.32±895.36	1474.13±848.72	0.537	0.587[b]	1352.17±721.06	1265.03±730.37	0.345	0.731[c]
Lipids, g		48.49±26.66	42.69±33.99	56.66±48.91	0.660	0.520[b]	53.14±36.28	44.96±30.22	0.915	0.363[c]
Proteins, g		60.58±25.81	55.89±35.4	73.67±40.37	1.244	0.294[b]	65.12±31.47	58.99±31.17	0.655	0.514[c]
HCO, g		156.53±84.05	148.14±136.56	168.08±74.34	0.150	0.861[b]	155.09±89.35	157.05±104.36	-0.227	0.821[c]
Fiber, g		11.46±7.88	9.28±6.37	9.7±6.14	0.700	0.500[b]	10.41±7.52	10.73±7.1	-0.310	0.758[c]
Iron, mg		8.75±4.74	7.86±7.14	9.13±4.95	0.241	0.787[b]	9.75±6.02	7.77±4.88	1.420	0.160[c]

Sodium, mg	1081.53±989.56	1019.34±1082.26	1623.53±1622.59	1.246	0.294[b]	1084.57±1312.49	1203.18±1014.16	-0.535	0.594[c]
Potassium, mg	752.45±792.19	514.72±502.69	1024.65±742.98	1.869	0.162[b]	818.07±847.59	677.59±638.26	0.724	0.472[c]
Calcium mg	558.75±328.89	536.13±596.16	627.39±570.55	0.156	0.856[b]	510.96±369.38	601.25±497.05	-0.956	0.342[c]
Phosphorus, mg	27.44±54.54	29.61±67.22	15.35±37.18	0.271	0.763[b]	26.98±52.56	25.3±57.81	0.079	0.937[c]
Sugar, g	33.35±32.67	17.48±22.33	40.28±35.7	2.468	0.092[b]	36.05±33.09	26.38±30.1	1.213	0.229[c]
Vitamin A, µg	359.32±396.57	319.54±301.04	387.11±229.5	0.149	0.862[b]	312.23±244.63	382.35±404.53	-0.938	0.351[c]
Vitamin B9, µg	120.06±139.82	94.68±69.82	183.81±122.88	1.987	0.145[b]	146.87±145.41	107.93±106.38	1.231	0.222[c]
Vitamin C, mg	86.06±143.35	69.51±114.63	112.1±118.01	0.379	0.686[b]	111.33±151.68	68.38±113.99	1.320	0.191[c]
Selenium, µg	26.64±23.19	31.58±25.24	38.67±21.66	1.288	0.282[b]	33.06±23.85	27.7±23.45	0.836	0.406[c]
Cholesterol, mg	155.93±105.31	166.84±177.18	240.19±202.19	1.599	0.209[b]	191.13±150.72	159.71±143.13	0.791	0.432[c]
SFA, g	3.46±4.69	1.92±3.8	2.98±5.45	0.725	0.488[b]	3.74±5.44	2.45±3.89	1.122	0.266[c]
MFA, g	6.13±6.93	3.61±8.64	5.86±7.38	0.769	0.468[b]	7.55±7.66	3.95±7.01	2.004	0.049[c]
PFA, g	2.69±3.45	1.63±3.93	2.41±3.06	0.589	0.558[b]	3.3±3.62	1.71±3.31	1.874	0.065[c]
GI	395.94±269.86	317.44±260.22	479.22±326.53	1.282	0.284[b]	419.17±309.85	368.28±255.62	0.645	0.521[c]
GL	96.01±69.35	95.38±80.42	108.86±90.34	0.149	0.862[b]	97.03±77.98	98.6±73.79	-0.209	0.835[c]
Milk, times/month	21.02±9.92	20.63±10.09	17.5±11.35	0.567	0.570[b]	20.13±10.52	20.49±9.99	-0.072	0.943[c]
Yogurt, times/month	10.93±8.74	7.16±4.68	10.17±9.24	1.471	0.237[b]	8.83±8.03	10.51±8.07	-0.848	0.399[c]
Chocolate, times/month	5.83±7.64	5.11±4.68	5.92±7.13	0.083	0.921[b]	5.27±7.41	5.93±6.46	-0.489	0.626[c]
Puffed cereals, times/month	10.05±10.79	14.16±12.81	10.25±11.25	0.893	0.414[b]	10.4±11.45	11.67±11.47	-0.326	0.746[c]
Cookies Maria style, times/month	4.07±5.82	6.47±10.01	3.17±3.76	1.075	0.347[b]	5.1±7.8	4.16±6.31	0.502	0.617[c]
Choco-cream cookies, times/month	4.69±6.3	3.89±6.64	3.42±3.96	0.254	0.776[b]	3.6±4.89	4.74±6.72	-0.869	0.388[c]
Muffins, times/month	8.67±9.97	12±12.07	9.67±10.71	0.639	0.531[b]	9.43±10.27	9.88±10.96	-0.268	0.789[c]
Donuts and croissants, times/month	3.31±6.38	2.32±4.44	3.83±8.01	0.255	0.775[b]	4.57±8.07	2.14±4.23	1.619	0.110[c]
Salad, times/month	9.93±8.9	9.68±7.7	15.5±10.09	2.064	0.135[b]	7.83±7.9	12.84±9.13	-2.412	0.018[c]
Spinach, swiss card, times/month	4.64±6.34	3.79±6.73	11.58±10.35	5.093	0.009[b]	5.9±7.5	5.33±7.77	0.400	0.691[c]
Vegetables on the side, times/month	5.69±7.06	5.37±7.24	9.17±10.1	1.105	0.337[b]	6.37±7.95	6.05±7.56	0.095	0.924[c]
Potatoes, times/month	4.4±4.89	5.63±4.51	10±8.39	4.818	0.011[b]	4.5±4.71	6.44±6.37	-1.527	0.131[c]
Legumes, times/month	9.52±8.97	9.89±9.18	7.17±7.7	0.404	0.669[b]	8.8±8.71	9.53±8.89	-0.409	0.683[c]
Rice, times/month	11.43±9.39	13.26±8.95	10.75±9.72	0.343	0.711[b]	13.3±9.88	10.74±8.74	1.083	0.283[c]
Pasta, times/month	10.14±8.36	12.26±8.82	12.25±11.42	0.490	0.615[b]	11.2±9.16	10.93±8.92	0.015	0.988[c]
Creamy soups, times/month	5.05±7.05	4±4.33	8.25±9.44	1.451	0.241[b]	6±8.37	4.81±5.86	0.693	0.491[c]
Eggs, times/month	8.17±7.45	8.84±7.57	8.5±5.27	0.059	0.942[b]	9.77±7.8	7.44±6.47	1.325	0.189[c]
Chicken or turkey, times/month	11.79±7.87	9.47±5.69	17±8.38	3.803	0.027[b]	12.37±8.65	11.81±7.14	0.297	0.767[c]
Beef, pig or lamb, times/month	7.67±5.82	7.95±5.99	6.67±5.99	0.186	0.831[b]	8.27±6.27	7.09±5.52	0.923	0.359[c]
Minced meat, times/month	3.36±3.82	3.11±2.75	3.25±4.67	0.030	0.970[b]	2.43±2.5	3.86±4.25	-1.750	0.085[c]
White fish, times/month	1.95±3.32	1.21±1.99	2±2.41	0.476	0.623[b]	1.6±3.29	1.88±2.58	-0.404	0.688[c]
Blue fish, times/month	1.02±2.2	1.26±1.76	3.08±6.72	1.875	0.161[b]	2.33±4.84	0.79±1.28	1.949	0.055[c]
Seafood, times/month	0.88±1.42	0.53±0.7	0.75±1.54	0.492	0.614[b]	0.8±1.4	0.74±1.22	0.199	0.843[c]
Croquettes, times/month	2.24±3.86	1.16±1.46	2.17±2.33	0.790	0.458[b]	1.57±3.05	2.21±3.26	-0.912	0.365[c]
Bread, times/month	5.55±7.36	7.05±8.23	9.25±9.88	1.043	0.358[b]	6.33±6.86	6.7±8.84	-0.270	0.788[c]
Ham, times/month	8.33±7.34	11.58±8.1	9.08±8.92	1.137	0.327[b]	8.9±7.1	9.58±8.36	-0.380	0.705[c]
White cheese, times/month	8.55±8.03	10.79±8.16	13.25±10.63	1.560	0.217[b]	9.27±8.33	10.35±8.83	-0.548	0.585[c]
Other cheese, times/month	3.88±7.01	4.84±8.17	5.5±10.19	0.237	0.789[b]	4.47±7.68	4.35±7.98	0.008	0.994[c]
Citric fruits, times/month	14.43±10.05	19.21±10.28	17.75±9.71	1.646	0.200[b]	13.6±9.6	18.05±10.22	-1.766	0.082[c]

Other fruits, times/month	17.74±12.5	21.47±8.97	26.67±4.62	3.408	0.039[b]	15.77±11.21	23.26±10.04	-3.031	0.003[c]
Canned fruit, times/month	0.64±1.12	1.84±6.41	0.83±1.4	0.826	0.442[b]	0.83±1.34	1.09±4.3	-0.347	0.729[c]
Natural fruit juice, times/month	8.24±9.33	8.68±10.73	9.33±9.65	0.062	0.940[b]	7.8±9.43	9.05±9.85	-0.351	0.727[c]
Commercial fruit juice, times/month	4.14±6.13	8.42±9.48	1.67±3.17	4.119	0.020[b]	4.1±7.05	5.37±7.21	-0.759	0.451[c]
Nuts, times/month	3.67±5.43	3.26±5.78	6±8.73	0.829	0.441[b]	3.47±6	4.28±6.28	-0.618	0.539[c]
Dairy desserts, times/month	2.95±4.16	2.89±4.93	5.92±9.13	1.513	0.227[b]	4±6.41	3.02±4.75	0.687	0.495[c]
Cakes, times/month	1.14±2.59	1.47±2.12	1.58±2.54	0.211	0.810[b]	1.2±2.67	1.37±2.3	-0.347	0.730[c]
Fry bags, times/month	5.6±7.72	5.37±5.85	2.92±4.14	0.742	0.480[b]	5.57±7.03	4.77±6.68	0.534	0.595[c]
Candies, times/month	8.33±9.07	5.16±7.32	6.25±8.61	0.978	0.381[b]	8.57±9.79	6.19±7.59	1.089	0.280[c]
Ice-cream, times/month	1.88±3.63	4.95±7.78	3.92±3.65	2.655	0.077[b]	3.27±4.84	2.84±5.41	0.291	0.772[c]
Soda, times/month	6.67±8.66	12.84±10.46	6.67±10.42	3.007	0.056[b]	7.57±9.82	8.77±9.7	-0.326	0.746[c]
Light soda, times/month	0.1±0.48	0.32±0.82	1±3.46	1.741	0.183[b]	0.57±2.24	0.12±0.54	1.247	0.216[c]
Wine, times/month	0.12±0.63	0.16±0.69	0.25±0.62	0.194	0.824[b]	0.3±0.95	0.05±0.21	1.663	0.101[c]
Beer, times/month	0.74±1.87	0.16±0.37	1.42±2.57	1.882	0.160[b]	0.87±2.1	0.58±1.56	0.627	0.533[c]
Non-alcohol beer, times/month	0.1±0.62	0±0	0±0	0.363	0.697[b]	0.13±0.73	0±0	1.187	0.239[c]
Distilled drinks, times/month	0.24±0.91	0±0	0.25±0.62	0.754	0.474[b]	0.23±0.82	0.14±0.68	0.510	0.611[c]

Data are presented as n (percentage) or mean ± standard deviation. CDMX: Mexico City; ZMVM: Metropolitan Zone of the Valley of Mexico; LMP/USG : Last Menstrual Period/Ultrasonography; DTB: Diabetes; HTN: hypertension; TB: tuberculosis; OB: obesity; MS: Metabolic syndrome; Wkends: week-ends; Occas: occasionally; NNS: non-nutritive sweeteners; HCO: Carbohydrates; BMI: Body Mass Index; Kcal: Kilocalories; SFA: Saturated Fatty Acids; MFA: Monounsaturated Fatty Acids; PFA: Polyunsaturated Fatty Acids; GI; Glycemic Index; GL: Glycemic Load; [a] Chi-squared test; [b] NOVA; [c] t-test.

Table S2. Alpha summary. Alpha diversity indexes for each group of frequency of NNS consumption. KW: Kruskal-Wallis; AOV: Analysis of Variance.

Alpha.diversity.index	*Q1_mean*	*Q2_mean*	*Q3_mean*	*Q4_mean*	*test*	*statistic*	*p.value*
Observed	161.14 ± 73.22	177.18 ± 82.05	209.5 ± 133.21	158.89 ± 82.02	KW	1.81	0.61
Shannon	2.29 ± 0.71	2.48 ± 0.71	2.46 ± 0.83	2.39 ± 0.74	AOV	0.21	0.89
Simpson	0.7 ± 0.17	0.76 ± 0.15	0.73 ± 0.2	0.71 ± 0.18	KW	1.37	0.71
InvSimpson	4.62 ± 2.91	6.5 ± 5.28	5.49 ± 3.13	4.63 ± 2.42	KW	1.37	0.71
Fisher	23.93 ± 10.66	26.48 ± 11.33	30.66 ± 17.93	25.42 ± 13.13	KW	1.35	0.72

Table S3. Kingdom statistics of the comparison between the quartiles of frequency of consumption of products with NNS with Kruskal-Wallis.

	Pr (>F)	*F*	*FDR*
Unassigned	0.40840785	0.97769198	0.40840785
d__Archaea	0.12605738	1.9732129	0.2595336
d__Bacteria	0.19009181	1.63123675	0.2595336
d__Eukaryota	0.1946502	1.61139389	0.2595336

nutrients MDPI

Table S4. Phylum statistics of the comparison between the quartiles of frequency of consumption of products with NNS with Kruskal-Wallis.

	Pr (>F)	*F*	*FDR*
Actinobacteriota	0.29109759	1.27124905	0.51539642
Bacteroidota	0.38654732	1.02606329	0.51539642
Firmicutes	0.66359437	0.52940091	0.66359437
Proteobacteria	0.29304488	1.26554984	0.51539642

Table S5. Genus statistics of the comparison between the quartiles of frequency of consumption of products with NNS with Kruskal-Wallis.

	Pr (>F)	*F*	*FDR*
Actinobacillus	0.38303689	1.03405487	0.73067108
Bifidobacterium	0.09095245	2.24331075	0.3638098
Blautia	0.79680374	0.33951813	0.79680374
Corynebacterium	0.07275411	2.42771289	0.3638098
Cutibacterium	0.72848935	0.43520274	0.79471566
Gemella	0.44266289	0.90625033	0.73067108
Methyloversatilis	0.17432186	1.70362743	0.52296559
Microbacterium	0.49431853	0.80691192	0.73067108
Prevotella	0.46679287	0.85870789	0.73067108
Sphingomonas	0.00469449	4.72101401	0.05633385
Staphylococcus	0.54800331	0.71222346	0.73067108
Streptococcus	0.63489556	0.57267278	0.76187468

nutrients

MDPI

Table S6. Microbiome data analysis using DESeq2 to compare the quartiles of frequency of consumption of products with NNS.

Base Mean	*log2Fold Change*	*lfcSE*	*stat*	*p*	*padj*	*Kingdom*	*Phylum*	*Class*	*Order*	*Family*	*Genus*	*Species*	*Group comparison*	*NNS_Group*
13.45	-23.49	2.99	-7.85	0.00	0.00	d__++Archaea	Euryarchaeota	Methanobacteria	Methanobacteriales	Methanobacteriaceae	Methanobrevibacter	NA	Q1 vs Q2	Q2
26.02	17.70	3.01	5.88	0.00	0.00	d__Bacteria	Firmicutes	Bacilli	Staphylococcales	Staphylococcaceae	*Staphylococcus*	Staphylococcus lugdunensis	Q1 vs Q2	Q1
16.73	-12.60	1.47	-8.55	0.00	0.00	d__Archaea	Euryarchaeota	Methanobacteria	Methanobacteriales	Methanobacteriaceae	*Methanobrevibacter*	NA	Q1 vs Q3	Q3
6.97	-8.31	0.99	-8.43	0.00	0.00	d__Bacteria	Cyanobacteria	Cyanobacteriia	Chloroplast	Chloroplast	Chloroplast	NA	Q1 vs Q4	Q4
854.79	-0.96	0.24	-4.06	0.00	0.04	d__Bacteria	Firmicutes	Bacilli	Lactobacillales	Streptococcaceae	*Streptococcus*	NA	Q1 vs Q4	Q4
4.80	-13.46	2.32	-5.80	0.00	0.00	d__Eukaryota	Vertebrata	Mammalia	Mammalia	Mammalia	Mammalia	Homo sapiens	Q2 vs Q3	Q3
1.84	-14.14	2.95	-4.80	0.00	0.00	d__Bacteria	Firmicutes	Bacilli	Staphylococcales	Staphylococcaceae	*Staphylococcus*	Uncultured bacterium	Q2 vs Q3	Q3
3.47	-13.69	2.94	-4.65	0.00	0.00	d__Eukaryota	Vertebrata	Mammalia	Mammalia	Mammalia	Mammalia	Homo_sapiens	Q2 vs Q3	Q3
23.23	-11.99	1.47	-8.18	0.00	0.00	d__Bacteria	Firmicutes	Bacilli	Staphylococcales	Staphylococcaceae	*Staphylococcus*	Staphylococcus lugdunensis	Q2 vs Q4	Q4
8.93	-11.32	1.45	-7.81	0.00	0.00	d__Bacteria	Firmicutes	Bacilli	Lactobacillales	Streptococcaceae	*Streptococcus*	Uncultured bacterium	Q2 vs Q4	Q4
3.07	-10.67	1.47	-7.28	0.00	0.00	d__Eukaryota	Vertebrata	Mammalia	Mammalia	Mammalia	Mammalia	Homo sapiens	Q2 vs Q4	Q4
